# Lysophosphatidic acid via LPA-receptor 5/protein kinase D-dependent pathways induces a motile and pro-inflammatory microglial phenotype

**DOI:** 10.1186/s12974-017-1024-1

**Published:** 2017-12-19

**Authors:** I. Plastira, E. Bernhart, M. Goeritzer, T. DeVaney, H. Reicher, A. Hammer, B. Lohberger, A. Wintersperger, B. Zucol, W. F. Graier, D. Kratky, E. Malle, W. Sattler

**Affiliations:** 1 Institute of Molecular Biology and Biochemistry, Medical University of Graz, Neue Stiftingtalstrasse 6/6, 8010 Graz, Austria; 2 BioTechMed-Graz, Graz, Austria; 3 Institute of Biophysics, Medical University of Graz, Graz, Austria; 4 Institute of Cell Biology, Histology and Embryology, Medical University of Graz, Graz, Austria; 5 Department of Orthopedic Surgery, Medical University of Graz, Graz, Austria

**Keywords:** Bioactive lipids, LPAR5, PKDs, Signal transduction, Migration, Pro-inflammatory mediators

## Abstract

**Background:**

Extracellular lysophosphatidic acid (LPA) species transmit signals via six different G protein-coupled receptors (LPAR1–6) and are indispensible for brain development and function of the nervous system. However, under neuroinflammatory conditions or brain damage, LPA levels increase, thereby inducing signaling cascades that counteract brain function. We describe a critical role for 1-oleyl-2-hydroxy-sn-glycero-3-phosphate (termed “LPA” throughout our study) in mediating a motile and pro-inflammatory microglial phenotype via LPAR5 that couples to protein kinase D (PKD)-mediated pathways.

**Methods:**

Using the xCELLigence system and time-lapse microscopy, we investigated the migrational response of microglial cells. Different M1 and M2 markers were analyzed by confocal microscopy, flow cytometry, and immunoblotting. Using qPCR and ELISA, we studied the expression of migratory genes and quantitated the secretion of pro-inflammatory cytokines and chemokines, respectively. Different transcription factors that promote the regulation of pro-inflammatory genes were analyzed by western blot. Reactive oxygen species (ROS) and nitric oxide (NO) production, phagocytosis, and microglial cytotoxicity were determined using commercially available assay kits.

**Results:**

LPA induces MAPK family and AKT activation and pro-inflammatory transcription factors’ phosphorylation (NF-κB, c-Jun, STAT1, and STAT3) that were inhibited by both LPAR5 and PKD family antagonists. LPA increases migratory capacity, induces secretion of pro-inflammatory cytokines and chemokines and expression of M1 markers, enhances production of ROS and NO by microglia, and augments cytotoxicity of microglial cell-conditioned medium towards neurons. The PKD family inhibitor blunted all of these effects. We propose that interference with this signaling axis could aid in the development of new therapeutic approaches to control neuroinflammation under conditions of overshooting LPA production.

**Conclusions:**

In the present study, we show that inflammatory LPA levels increased the migratory response of microglia and promoted a pro-inflammatory phenotype via the LPAR5/PKD axis. Interference with this signaling axis reduced microglial migration, blunted microglial cytotoxicity, and abrogated the expression and secretion of pro-inflammatory mediators.

**Electronic supplementary material:**

The online version of this article (10.1186/s12974-017-1024-1) contains supplementary material, which is available to authorized users.

## Background

Microglia are resident immune cells of the central nervous system (CNS) [1] and are endowed with specific receptor sets that are able to detect subtle alterations of the finely tuned micromilieu in the CNS [2]. Even in the resting state, the dynamic microglial processes scan the CNS environment and respond to danger signals [3]. Microglia have been acknowledged to be key players under both physiological and pathological conditions [4]. They have diverse roles in the healthy brain, from sculpting developing neuronal circuits to guiding learning-associated plasticity, and numerous studies provide insights into their involvement in CNS disorders [5]. Neuronal injury results in the release of ATP, neurotransmitters, growth factors, cytokines, and changes in local ion homeostasis inducing microglial activation [6].

Microglia regulate multiple aspects of inflammation, such as repair, regeneration, cytotoxicity, and immunosuppression, depending on their different activation states or phenotypes [7]. Depending on the signal encountered, microglia can activate various programs that determine the severity of the response [8]. These responses involve directed migration to the site of injury and subsequent release of numerous inflammatory mediators [9]. Within the simplified M1/M2 dichotomy, polarized M1 microglia produce pro-inflammatory cytokines and neurotoxic molecules, which contribute to dysfunction of neural network and promote inflammation, whereas polarized M2 microglia secrete anti-inflammatory mediators and neurotrophic factors that are involved in restoring homeostasis [7]. These differential responses are indicative of the ability of microglia to promote neuronal survival or degeneration [10].

However, the situation is not clear-cut and the validity of the M1/M2 concept has been questioned [11]. Interestingly, some studies show that increased microglial activity can have controversial effects on disease pathology [12]. The mechanisms for microglial activation and their potential contributions to neuronal degeneration are a matter of debate, and knowledge regarding the molecular diversity of microglia in different disease settings is growing [4]. Recent studies have identified a novel microglial phenotype associated with neurodegenerative diseases [13] and uncovered pathways that regulate microglial functional phenotype in neurodegeneration [14].

In response to pathological stimuli, microglia exhibit morphological changes and migrate towards the lesion site. Cell migration can be triggered by a diverse array of chemoattractants including peptides and proteins (e.g., chemokines), small hydrophilic molecules (e.g., nucleotides), and bioactive lipids. Among the latter class, lysophosphatidic acid (LPA) species have the capacity to act as extracellular signaling molecules by activation of downstream cascades via six different G protein-coupled receptors (GPCRs termed LPAR1–6) [15, 16]. These GPCRs couple to one or more of the four Gα proteins (G_i/o_, G_12/13_, G_q_, and G_s_) that initiate downstream signaling cascades [16]. The CNS is under control of these pathways since LPA displays profound effects on brain capillary endothelial cells, neurons, and glial cells [17]. LPA induces the disruption of junctional complexes of brain endothelial cells [18, 19], rapid growth cone collapse, and neurite retraction of mature neurons [20]. Mice lacking the *Lpa1* gene show craniofacial defects and perinatal lethality due to impaired suckling behavior [21] and develop a fetal hydrocephalus [22].

Several studies have suggested that glial cells are important target cells for LPA [23–25]. Rodent and human microglial cell lines express LPARs and respond to LPA [26, 27]. In the murine BV-2 microglial cell line, LPA elicits membrane hyperpolarization due to an activation of Ca^2+^-dependent K^+^ currents [28] and Ca^2+^-activated K^+^ channels are a requirement for LPA-dependent induction of microglial migration [29]. In addition to ion homeostasis, LPA controls microglial activation and energy homeostasis (human C13NJ cells) [27], modulates oxidative stress response (murine BV-2 cell line) [30], regulates the induction of chronic pain (in vivo and primary murine microglia) [31], and interferes with pro-inflammatory cytokine production (BV-2) [32].

Generally, under physiological conditions, LPA-mediated signaling contributes to normal development and function of the CNS. However, in response to injury, LPA levels rise significantly in the brain and cerebrospinal fluid (CSF) [22, 33–36]. LPA levels are elevated in the human (0.05 controls vs. 0.27 μM post injury) and mouse (0.8 and 2 μM, prior vs. post injury) CSF in response to traumatic brain injury [37]. LPA signaling initiates neuropathic pain [38], where LPAR1 [39] and LPAR5 [40] contribute via independent mechanisms. Findings that LPAR5 is activated during nerve injury (but not under basal conditions) are consistent with the fact that LPA levels rise significantly in response to spinal cord injury [35, 36]. Demyelination in the injured spinal cord was (at least in part) ascribed to LPA-activated microglia [36]. Lysophosphatidylcholine injected intrathecally is converted to LPA via autotaxin (ATX)-mediated pathways and, in an LPAR3-dependent feed-forward loop, induces further endogenous synthesis of LPA [41]. It was suggested that within this setting, microglial activation is responsible for de novo LPA synthesis and concomitant development of neuropathic pain [42]. We have recently reported that LPAR5 transmits pro-inflammatory signals in murine BV-2 and neonatal primary murine microglia (PMM) [43].

Many of the phenotypic responses of microglia towards LPA depend on intracellular phosphorylation events. LPA-mediated pathways activate protein kinase D isoforms (PKD1–3) that are classified within the calcium/calmodulin-dependent protein kinase superfamily [44]. Among a multitude of cellular functions, PKD members regulate directed cell migration by controlling anterograde membrane trafficking [45] or by directly affecting actin organization at the leading edge [46, 47] and are important constituents of the secretory machinery [48]. In addition, PKD isoforms play an important role in inflammatory responses [49]. In a variety of cells, PKD induces NF-κB activation via GPCR agonists or oxidative stress [50–52]. Moreover, PKD1 has been reported to mediate hyperalgesia and maintain inflammatory heat hypersensitivity [53].

Because our previous study revealed that BV-2 and PMM express high levels of LPAR5 [30], we elucidated its role in microglial plasticity. Members of the PKD family are activated by GPCR ligands, including LPARs, and mediate an inflammatory response in the CNS [54]. Therefore, we hypothesized that LPAR5 downstream activation of the PKD pathway couples to LPA-mediated signaling events in microglia.

## Methods

### Materials

The cell culture medium RPMI 1640 and Dulbecco’s modified Eagle’s medium (DMEM), fetal calf serum (FCS), antibiotics, and trypsin were obtained from Invitrogen (Waltham, MA, USA). LPA (1-oleoyl-2-hydroxy-*sn*-glycero-3-phosphate; LPA18:1) was from Sigma-Aldrich (St. Louis, MO, USA). The pharmacological LPAR5 antagonist [5-(3-chloro-4-cyclohexylphenyl)-1-(3-methoxyphenyl)-1*H*-pyrazole-3-carboxylic acid] (TCLPA5) was from Echelon Tocris (Bristol, UK). CRT0066101, a PKD family antagonist, was a generous gift from Dr. Christopher Ireson (Cancer Research Technology, London, UK). Anti-PKD_1_ PKD1 and anti-phospho-PKD1 (pPKD1, Ser^744/748^) rabbit antibodies were from Cell Signaling (Beverly, MA, USA), anti-PKD2  and anti- pPKD2 (Ser^848^) rabbit antibodies were from Abcam (Cambridge, UK), and mouse anti- PKD3 antibody was from Santa Cruz (San Diego, CA, USA). Antibodies against cyclooxygenase-2 (COX-2) and arginase-1 (Arg-1; used only for western blotting) were from Cell Signaling (Beverly, MA, USA), and inducible nitric oxide synthase (iNOS) antibody was from BD Biosciences (San Jose, CA, USA). For immunofluorescence, the COX-2 and Arg-1 antibodies were from Santa Cruz (Dallas, TX, USA), and the antibodies against RELMα (FIZZ-1) and iNOS were from Abcam (Cambridge, UK). Anti-ionized calcium-binding adapter molecule 1 (Iba-1) was from Wako Chemicals (Neuss, Germany), and the CD11b antibody was from Novus Biologicals (Littleton, CO, USA). PE-CD40, APC-CD86, and PE-CD206 antibodies and their isotype controls were from e-Bioscience (San Diego, CA, USA). Antibodies against non-phosphorylated and phosphorylated mitogen-activated protein kinases ERK1/ERK2, p38 MAPK, JNK, and AKT, p65-NF-κB, c-Jun, STAT1, and STAT3 were from Cell Signaling (Beverly, MA, USA). Cyanine (Cy-2/Cy-3)-labeled antibodies were from GE Healthcare (Vienna, Austria). MISSION lentiviral transduction particles (shPKD1 and shPKD2), MISSION non-mammalian short hairpin RNA (shRNA) control transduction particles, poly-d-lysine (PDL) hydrobromide, FITC-conjugated tomato lectin, monoclonal anti-mouse β-actin (clone AC-74), and β-tubulin antibodies were from Sigma-Aldrich (St. Louis, MO, USA). All primers and kits used in qRT-PCR were from Qiagen (Hilden, Germany).

### BV-2 culture

The murine microglial cell line BV-2 was from Banca Biologica e Cell Factory (Genoa, Italy). Cells were grown and maintained in the RPMI 1640 medium supplemented with 10% FCS, 1% penicillin, 1% streptomycin, and 10 ml l-glutamine (200 mM) at 37 °C in a humidified incubator under 5% CO_2_ and 95% air. The culture medium was changed to fresh medium every 2 or 3 days. When cells reached confluence, cells were split or used for experiments.

### Primary microglial culture

PMM were isolated and purified from C57BL/6 cortices of neonatal (P0–P4) mice as previously described [43]. In brief, the brain cortices were isolated from the whole brain, stripped from their meninges, and minced with scissors into small pieces. Glial cells were separated by trypsinization (0.1% trypsin, 20 min, 37 °C, 5% CO_2_), and the cell suspension was cultured in 75-cm^2^ tissue culture flasks precoated with 5 mg/ml PDL in DMEM containing 15% FCS, 1% penicillin/streptomycin, and 10 ml l-glutamine. After 3 days in culture, the medium was changed to fresh DMEM containing 10% FCS and cells were cultured for another 10 to 14 days. Microglia were removed from the mixed glial cell cultures by smacking the culture flasks 10–20 times and seeded onto PDL-coated cell culture plates for future use. The purity of PMM was determined by immunocytochemistry (using CD11b immunostaining or tomato lectin staining) and was always > 95%.

### CATH.a neuron culture

Murine neuronal CATH.a cells (ATCC) were grown and maintained in the RPMI 1640 medium supplemented 10% horse serum, 5% FCS, 1% penicillin/streptomycin, 0.4% HEPES, and 0.2% sodium pyruvate at 37 °C in a humidified incubator under 5% CO_2_ and 95% air. The culture was maintained by transferring floating cells to additional flasks. When cells reached confluence, they were split into new flasks (subcultivation ratio of 1:4) using 0.12% trypsin without EDTA or used immediately for the experiments.

### LPA treatment

Cells (BV-2 or PMM) were plated in 6-, 12-, or 24-well plates (PDL-coated in case of primary microglia) and allowed to adhere for 2–3 days. Cells were always incubated in serum-free medium overnight before the medium was changed to serum-free RPMI (BV-2) or DMEM (PMM) containing 0.1% fatty acid-free bovine serum albumin (BSA; control) or DMEM containing 0.1% BSA or LPA (1 μM). BSA was used as an LPA carrier. Aqueous LPA stock solutions (5 mM) were stored at − 70 °C. Only freshly thawed stocks were used for the experiments.

### Treatments with pharmacological inhibitors

TCLPA5 is a specific inhibitor for LPAR5 [55], and CRT0066101 [56] is a PKD family inhibitor. CRT0066101 exhibits high selectivity for PKDs not interfering with the activity of a panel of > 90 protein kinases, including PKCα, MEK, ERK, c-Raf, and c-Src [56]. Both inhibitors were diluted in DMSO (stock concentrations 100 and 10 mM, respectively) and kept at − 20 °C. TCLPA5 solutions are stated to be stable at − 20 °C for a maximum of 40 days. During the experiments, TCLPA5 and CRT0066101 were used at a final concentration of 5 and 1 μM, respectively. Cells were preincubated with the antagonists as stated for each experiment.

### Immunoblotting

BV-2 cells (seeded onto six-well plates at a density of 1 × 10^5^ cells/well) or PMM (cultured on PDL-coated 12-well plates at a density of 5 × 10^5^ cells/well) were used for analyses of PKD isoforms’ expression and the phosphorylation state of PKDs, JNK, AKT, ERK1/ERK2, and p38 MAPK, p65-NF-κB, c-Jun, STAT1, and STAT3. Culture medium was removed, and cells were washed twice with ice-cold PBS. The cells were lysed in RIPA buffer (50 mM Tris-HCl (pH 7.4), 1% NP-40, 150 mM NaCl, 1 mM Na_3_VO_4_, 1 mM NaF, 1 mM EDTA) containing protease inhibitors (aprotinin, leupeptin, pepstatin, 1 μg/ml each; Sigma-Aldrich), 10 μM PMSF, and phosphatase inhibitor cocktail (Thermo Scientific, Waltham, MA, USA) and then scraped and centrifuged at 13,000 rpm for 10 min. Protein content was determined using the BCA kit (Thermo Scientific) and BSA as standard. Protein samples (100 μg) were separated on 10% SDS-PAGE gels and transferred to polyvinylidene difluoride membranes. Membranes were blocked with 5% low-fat milk in Tris-buffered saline containing Tween 20 (TBST) for 2 h at room temperature (RT) and incubated with the primary antibodies overnight with gentle shaking at 4 °C. After removal of primary antibodies, the membranes were washed for 30 min in TBST and incubated for 2 h at RT with anti-rabbit (1:10,000) or anti-mouse (1:5000) as secondary antibodies. Following three washing steps with TBST for 1 h, immunoreactive bands were visualized using ECL or ECL plus reagents and detected with a chemiluminescence detection system (ChemiDoc; Bio-Rad, Berkeley, CA, USA). In some cases, the membranes were stripped using a stripping buffer (140 μl β-mercaptoethanol in 20 ml of 60 mM Tris/2% SDS (pH 6.8) buffer) under gentle shaking for 30 min at 50 °C in a water bath, washed for 1 h in TBST, blocked with 5% low-fat milk in TBST for 1 h at room temperature, and probed with the pan antibodies for PKD1, PKD2, JNK, AKT, ERK1/ERK2, and p38 MAPK, p65-NF-κB, c-Jun, STAT1, and STAT3. β-actin or β-tubulin was used as loading controls.

### Immunofluorescence

Double immunofluorescence was carried out in BV-2 and PMM. Cells were seeded in chamber slides (PDL-coated in case of primary cells) at a density of 1.5 × 10^4^ and 3 × 10^4^ cells/well, respectively. Cells were serum-starved overnight and incubated in the absence or presence of LPA or LPA plus inhibitors as indicated. Cells were washed with prewarmed PBS, fixed with paraformaldehyde (4% in 0.1 M PBS) for 15 min, and permeabilized with 0.5% Triton X-100/PBS for 10 min at 25 °C. Following three washing steps with PBS, cells were incubated with blocking buffer (Thermo Scientific, Waltham, MA, USA) for 1 h at 4 °C and incubated with the primary antibody (1:50) overnight at 4 °C. The slides were then washed with PBS and incubated with fluorescently labeled secondary antibody (1:200) for 30 min. All slides were washed three times with PBS stained with Hoechst 33342 (Invitrogen, Waltham, MA, USA) and mounted using a mounting medium (Dako, Vienna, Austria). Confocal fluorescence microscopy imaging was performed using a Leitz/Leica TCSSP2 microscope (Leica Lasertechnik GmbH, Heidelberg, Germany). Quantitation of fluorescence intensity and morphological analysis were performed with ImageJ. At least 50 cells out of three different areas per chamber were analyzed.

### Lentiviral transduction (shRNA)

PMM were cultured on PDL-coated 24-well plates at a density of 1.2 × 10^5^ cells/well. Polybrene (8 μg/ml) solution and viral particles (multiplicity of infection = 2) were added onto cultured microglia. We used shRNA control transduction lentiviral particles and PKD1-specific (shPKD1 NM_008858, clone ID: TRCN0000024007, sequence: *CCGGCCTTCAGCTTTAACTCCCGTTCTCGAGAACGGGAGTTAAAGCTGAAGGTTTTT*) and PKD2-specific (shPKD2 NM_178900, clone ID: TRCN0000322346, sequence: *CCGGGTACGACAAGATCCTGCTCTTCTCGAGAAGAGCAGGATCTTGTCGTACTTTTTG*) constructs. After 12 h of treatment with the shRNA control transduction particles and the PKD1 and PKD2 silencing constructs, the medium was replaced by a prewarmed conditioned medium prepared from mixed glial cultures after centrifugation and filtration through a 0.45-μm filter. Cells were kept at 37 °C/5% CO_2_ for 72 h and collected to validate silencing efficacy by qPCR or to proceed with the experiments described below.

### Time-lapse microscopy

BV-2 cells and PMM were seeded onto 24-well plates at a density of 2 × 10^4^ and 5 × 10^4^ cells/well, respectively. Cells were cultured in serum-free medium overnight and then treated with the indicated concentrations of LPA, with LPA plus DMSO (to account for vehicle effects), or with 1 μM LPA in the absence or presence of TCLPA5 (5 μM) or CRT0066101 (1 μM). Control cells were incubated in serum-free medium in the absence or presence of DMSO (negative controls) or treated with *N*-arachidonylglycine (NAGly; 1 μM; Sigma-Aldrich).

For silencing experiments, PMM (non-transduced or transduced with lentiviral particles containing sh-scrambled, shPKD1 or shPKD2) were cultured on PDL-coated 24-well plates at a density of 1.2 × 10^5^ cells/well. After transduction, the cells were incubated in serum-free medium in the absence or presence of LPA (1 μM).

Images were acquired every 20 min for 24 h at five different positions of each well using a Zeiss Cell Observer microscope. Data analysis was carried out using ImageJ. Image intensity correction was achieved by correcting each image median value to 125 (8-bit images have a resolution of 255-Gy levels). Image stabilization was achieved using the Lucas-Kanade method with a macro for ImageJ (K. Li, “The image stabilizer plugin for ImageJ” http://www.cs.cmu.edu/~kangli/code/Image_Stabilizer.html; ^®^Kang Li) to correct for changes in position due to mechanical tolerances in the microscope stage. Equalization of low-frequency variations in the background signal of the image using an FFT bandpass filtering and reducing low- and high-frequency changes in the images (low-frequency filter set to 40 pixels and high-frequency filter set to 6 pixels) enabled simple thresholding of the images. After thresholding, which allowed object selection, a Gaussian blur was applied. The resulting TIFF images were analyzed using ImageJ inherent functions. The ImageJ Manual Tracking plug-in was used to manually track the cells. In random, a minimum of 20 viable cells per condition was selected and followed. Using the ImageJ Chemotaxis and Migration Tool plug-in, the accumulated distance (the total cell path traveled by the cell), the Euclidean distance (the distance between the start and end points), and cell velocity were calculated. Experiments were repeated at least two times.

### xCELLigence migration assay

BV-2 cells were cultured in six-well plates at a density of 3 × 10^5^ cells/well and incubated in serum-free RPMI or pretreated with TCLPA5 (5 μM) or CRT0066101 (1 μM) for 3 h. Chemotaxis assays were carried out using CIM-16 well plates and an xCELLigence RTCA-DP instrument (Roche Diagnostics, West Sussex, UK). LPA solutions were prepared at the desired concentrations, and 160 μl of them was loaded in the lower wells of the CIM-16 plate. NAGly and serum-free medium served as positive and negative chemotaxis controls, respectively.

Following upper chamber attachment, the upper wells were first filled with 50 μl of prewarmed serum-free medium and the plate was left at RT for 30 min to pre-equilibrate. Cultured cells were trypsinized and resuspended in serum-free medium in the absence or presence of TCLPA5 (5 μM) or CRT0066101 (1 μM). Fifty microliters of the cell suspensions (containing 3 × 10^4^ cells) was placed into the upper wells. The plate was transferred to the RTCA-DP instrument, and data were collected every 5 min over 24 h. As cells pass through the pores of the filter with an embedded gold microelectrode, an increase in electrical impedance corresponds to increased numbers of migrating cells (*cell index*). Data were normalized and analyzed using the RTCA software 1.2.1. Experiments were performed at least three times in triplicate.

### qPCR analysis

BV-2 cells (24-well plates, 5 × 10^4^ cells/well) or PMM (PDL-coated 24-well plates, 2.5 × 10^5^ cells/well) were incubated in serum-free medium overnight and then treated with LPA (1 μM), LPA plus DMSO, LPA plus TCLPA5 (5 μM), or LPA plus CRT0066101 (1 μM). As negative controls, cells were incubated in serum-free medium in the presence of 0.1% BSA or DMSO. Total RNA was extracted using the RNeasy Mini or RNeasy Micro kit, respectively (Qiagen, Hilden, Germany), and quantitated using NanoDrop (Thermo Fisher Scientific, Waltham, MA, USA). RNA was reverse-transcribed using the SuperScript^®^ III reverse transcription kit (Invitrogen, Waltham, MA, USA). qPCR was performed on an Applied Biosystems 7900HT Fast Real-Time PCR System using the QuantiTect SYBR^®^ Green PCR kit (Qiagen, Hilden, Germany). Amplification of murine hypoxanthine-guanine phosphoribosyltransferase (HPRT) was performed on all samples as internal control for variations in messenger RNA (mRNA) concentration. Expression profiles and associated statistical parameters were analyzed using the relative expression software tool (REST; http://www.gene-quantification.de/rest-index.html) using a pairwise re-allocation test. Gene-specific primers were purchased from Qiagen, and the primer sequences of target genes are listed in Table 1.

**Table 1:** Primers used for real-time PCR analyses

Gene	Detected transcripts	Amplicon size
*Prkd1*	NM_008858 (3778 bp) XM_006515586 (3805 bp) XM_006515588 (4308 bp) XM_006515589 (3220 bp) XM_006515590 (3422 bp) XM_006515591 (2593 bp)	149
*Prkd2*	NM_001252458 (3340 bp) NM_178900 (3625 bp) XM_006539444 (2087 bp)	116
*Prkd3*	NM_001171004 (5887 bp) NM_001171005 (5083 bp) NM_029239 (5884 bp) XM_006525065 (5194 bp)	100
*Itga5*	NM_010577 (4397 bp)	136
*Itgav*	NM_008402 (7055 bp)	66
*Mmp9*	NM_013599 (3174 bp)	104
*Mmp14*	NM_008608 (2597 bp)	122
*Vasp*	NM_009499 (2267 bp)	88
*Wasf2*	NM_153423 (4088 bp)	129
*Vegfa*	NM_001025250 (3547 bp)	62

### ELISA

IL-1β, TNF-α, IL-6, CCL5 (RANTES), CXCL2 (MIP-2), and CXCL10 (IP-10) concentrations in the cellular supernatants were quantitated using the murine ELISA development kits (PeproTech, NJ, USA) [43]. Briefly, BV-2 and PMM were seeded in triplicate onto 12-well and PDL-coated 24-well plates at a density of 5 × 10^4^ and 2.5 × 10^5^ cells/well, respectively. After overnight serum starvation, cells were incubated in serum-free medium containing LPA (1 μM) in the absence or presence of the antagonists for the indicated time periods. For each time point, the supernatants were collected and kept at − 70 °C until further use. The assays were performed according to the manufacturer’s instructions. Standard curves for each ELISA were done in triplicates. The concentrations of the cytokines and chemokines were determined using the external standard curve.

### Total nitric oxide assay

iNOS activity was assessed indirectly using the total nitric oxide assay kit (Enzo Life Sciences, Switzerland). In this Griess assay, nitrate is reduced to nitrite by means of nitrate reductase. The accumulated total nitrate levels were measured in the supernatant of cells that were incubated in serum-free medium, containing LPA in the absence or presence of the antagonists for 24 or 48 h. Fifty microliters of the supernatant from each sample was processed according to the manufacturer’s protocol. The total nitrate concentration per sample was determined using the external calibration curve (0–100 μM nitrate).

### Measurement of carboxy-H_2_DCFDA oxidation

Intracellular reactive oxygen species (ROS) levels were measured using the DCFDA cellular ROS detection kit (Abcam, Cambridge, UK). After internalization and subsequent hydrolysis, the redox indicator probe carboxy-H_2_DCFDA is converted to carboxy-H_2_DCF, which, in the presence of oxidant species, is converted to fluorescent carboxy-DCF [57]. BV-2 cells were seeded in black clear-bottom 96-well plates at a density of 5 × 10^4^ cells/well [43]. Cells were allowed to adhere overnight and then incubated with 20 μM DCFDA for 40 min at 37 °C in the dark. The solution was removed, and the cells were incubated in serum-free medium, containing LPA in the absence or presence of the antagonists for 3 and 6 h. Fluorescence intensity was measured with excitation and emission wavelengths of 485 and 535 nm, respectively.

### Lactate dehydrogenase assay

Lactate dehydrogenase (LDH) activity was used as an indicator of cytotoxicity (Cayman Chemical, Ann Arbor, MI, USA) of CATH.a neurons.

BV-2 cells were seeded in triplicate onto 12-well plates at a density of 5 × 10^4^ cells/well. After overnight serum starvation, cells were incubated in serum-free medium, containing LPA in the absence or presence of the antagonists for the indicated time periods. For each time point, the supernatants were collected and kept at − 70 °C until further use.

The CATH.a neurons were seeded in a 96-well plate at a concentration of 1 × 10^5^ cells/well and allowed to adhere. Following overnight serum starvation, the cells were incubated with the supernatants collected from the abovementioned BV-2 cells. Three wells containing only the medium without cells were used for background control. In order to measure maximum and spontaneous release, cells were incubated with 10% Triton X-100 and assay buffer, respectively. Cells were kept at 37 °C (5% CO_2_) for 24 h, and then the plate was centrifuged at 1300 rpm for 5 min. One hundred microliters of the supernatants was transferred to a new 96-well plate, and 100 μl of LDH reaction solution was added to each well. The plate was incubated at 37 °C (5% CO_2_) for 30 min under gentle shaking, and the absorbance at 490 nm was measured using a plate reader.

### Statistical analysis

All experiments were performed using three or four replicates per experimental group and repeated three times (unless otherwise stated). For statistical analysis, data obtained from independent measurements are presented as mean + SD or mean + SEM as indicated in the figure legend. Statistical tests were performed using the GraphPad Prism (version 5.0a) for Mac (GraphPad Software, Inc., San Diego, CA, USA). Data were analyzed by one-way ANOVA followed by the Bonferroni post hoc test or unpaired Student’s *t* test. In the case of qPCR experiments, the expression profiles and associated statistical parameters were analyzed using the REST (http://www.gene-quantification.de/rest-index.html) using a pairwise re-allocation test. Values of *p* < 0.05 were considered significant, unless otherwise stated.

## Results

### LPA activates the MAPK and AKT pathways via LPAR5/PKD signaling in microglia

**Fig. 1:**
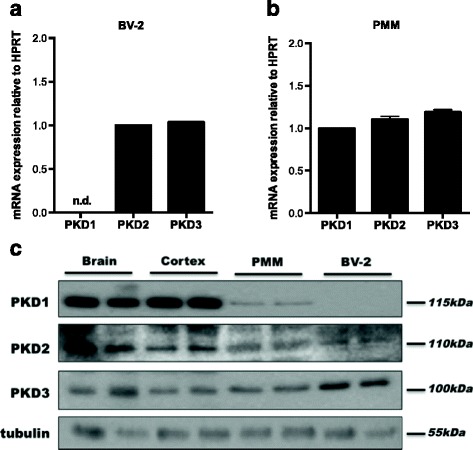
PKD isoform expression in BV-2 cells and PMM. Gene expression of PKD1–3 in **a** BV-2 and **b** PMM was analyzed by qPCR and normalized to HPRT. Values are expressed as mean + SD. PKD2 expression in BV-2 and PKD1 in PMM was arbitrarily set to 1. n.d. not detectable. **c** Protein expression of PKD isoforms was determined by western blotting. Samples from the whole brain and cortex were used as controls. One representative blot out of three is shown. β-Tubulin was used as loading control

PKD expression analysis in BV-2 and PMM by qPCR revealed that BV-2 cells express PKD2 and PKD3 (Fig. 1a; with mean Ct values: PKD2 25.8 and PKD3 24.9), while PMM express mRNA for all three PKD family members (Fig. 1b; mean Ct values: PKD1 29.0, PKD2 26.9, and PKD3 24.9). PKD1–3 protein was detected in mouse brain lysates, in the cortex, and in PMM. In line with mRNA data, protein expression levels of only PKD2 and PKD3 were detected in BV-2 cells (Fig. 1c).

In BV-2 cells, LPA induced phosphorylation of PKD2, JNK, AKT, ERK1/ERK2, and p38 MAPK (Additional file 1: Figure S1). Preincubation with TCLPA5 (an *LPAR5 inhibitor*) (0.5–8 h) prior to subsequent treatment with LPA (1 μM) in the presence of the inhibitor revealed that the antagonist suppressed activation of downstream signaling proteins (Additional file 1: Figure S1A) that regulate microglial function. Bar graphs in Additional file 1: Figure S1B represent densitometric evaluation of the western blots. Inhibition of PKD isoforms with CRT0066101 (a *PKD family inhibitor*) suppressed the LPA-induced activation of PKD2, JNK, AKT, ERK1/ERK2, and p38 MAPK (Additional file 2: Figure S2A). Densitometric evaluation is shown in Additional file 2: Figure S2B.

**Fig. 2:**
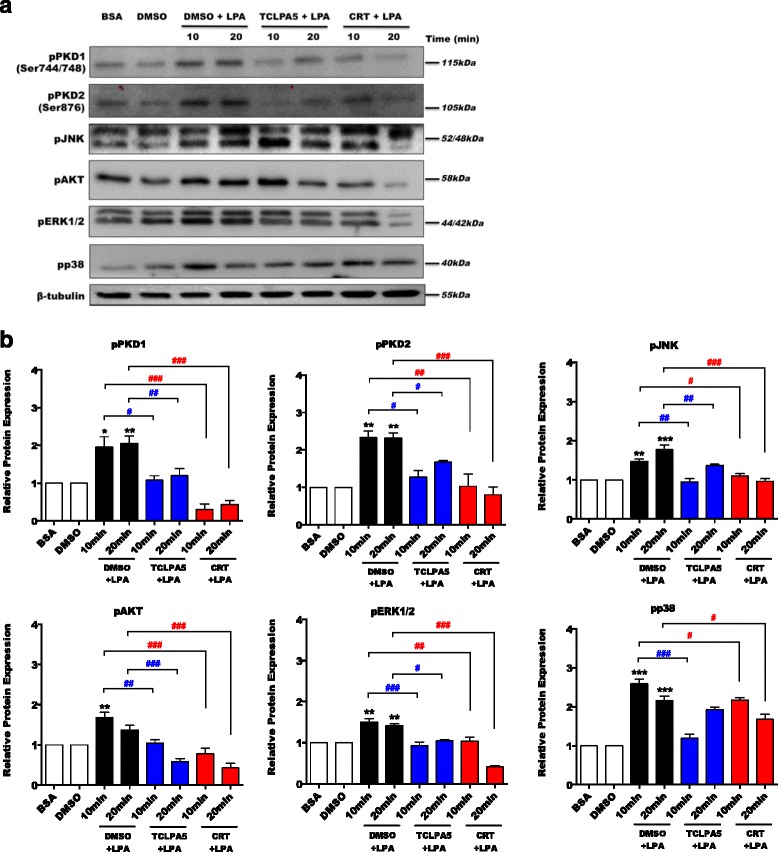
TCLPA5 and CRT0066101 (CRT) inhibit LPA-mediated downstream signaling. **a** PMM were preincubated overnight with TCLPA5 (5 μM) or CRT (1 μM) before treatment with LPA (1 μM) or LPA (1 μM) plus each inhibitor for the indicated times. Cells incubated with 0.1% BSA or DMSO were used as controls. The phosphorylation states of PKDs, JNK, AKT, ERK1/ERK2, and p38 were detected using western blotting. One representative blot is shown. **b** Densitometric analysis of western blots (*N* = 3). Results represent mean values + SEM (*p < 0.05, **p < 0.01, ***p < 0.001, compared to DMSO-treated cells; ^#^ *p* < 0.05, ^##^ *p* < 0.01, ^###^ *p* < 0.001, LPA plus TCLPA5 or CRT versus DMSO + LPA; one-way ANOVA with the Bonferroni correction)

In PMM, activation of PKD1 and PKD2 was observed after 10 min, reached a maximum after 20 min, and then decreased (Fig. 2). This PKD activation pattern correlated with the time course of JNK, AKT, ERK1/ERK2, and p38 MAPK phosphorylation (data not shown). In the presence of TCLPA5 or CRT0066101, phosphorylation of PKD1 and PKD2, JNK, AKT, ERK1/ERK2, and p38 MAPK was significantly attenuated (Fig. 2a). Bar graphs in Fig. 2b represent densitometric evaluation.

**Fig. 3:**
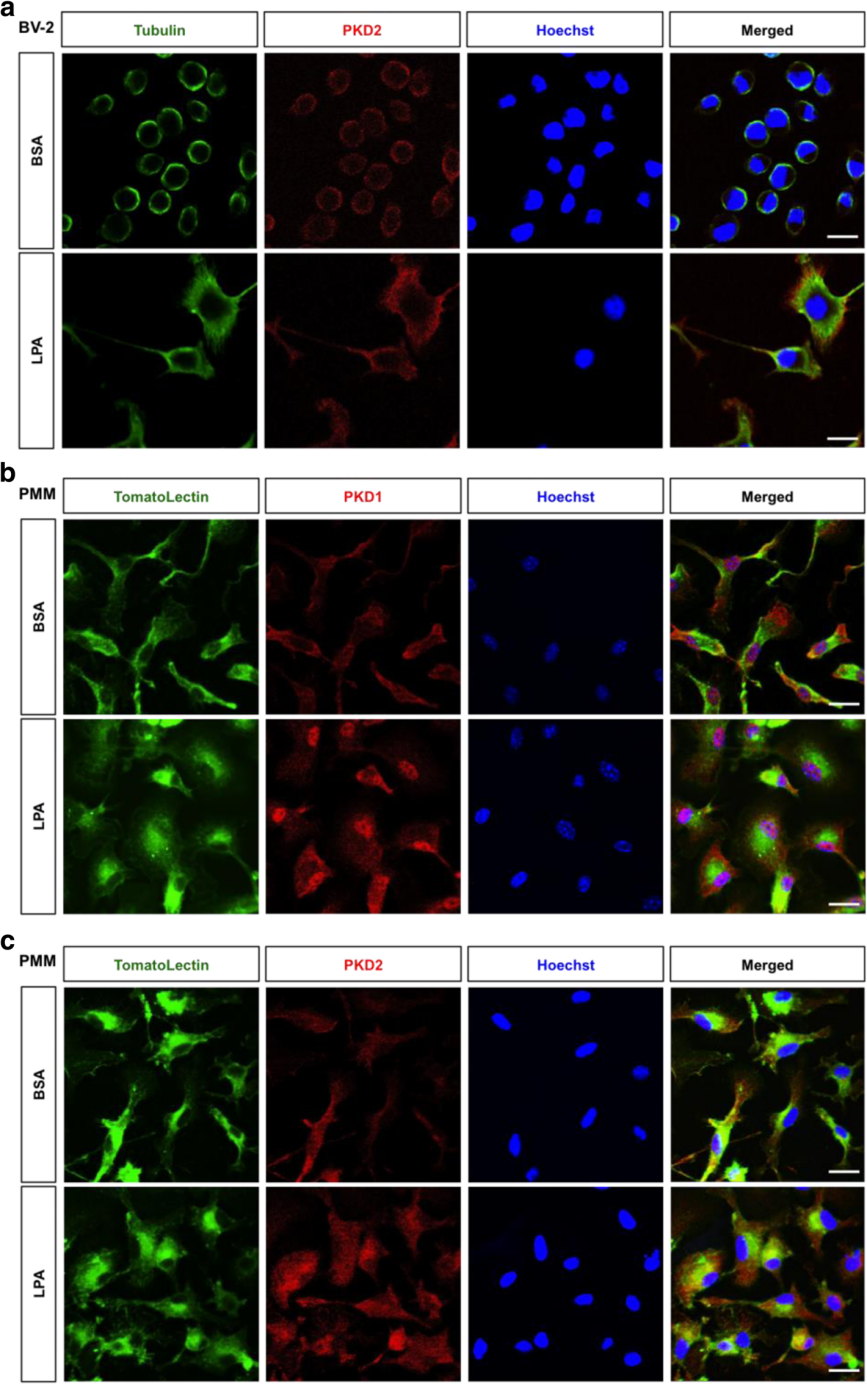
Intracellular trafficking of PKD1 and PKD2 in response to LPA. **a** BV-2 cells were cultured on chamber slides, serum-starved overnight, and incubated in the presence of 0.1% BSA (control) or LPA (1 μM) for 24 h. Cells were fixed, permeabilized, blocked, and stained for tubulin, PKD2, and nuclei (Hoechst). **b**, **c** PMM were cultured on chamber slides and serum-starved overnight. Cells were incubated in the presence of 0.1% BSA (control) or LPA (1 μM) for 24 h and stained with tomato lectin, **b** PKD1 or **c** PKD2 antibodies, and nuclei (Hoechst). Images were obtained using a Leica confocal microscope. Scale bars = 20 μm

PKDs translocate between different cellular compartments to be able to fulfill their functions in response to activation [58]. Therefore, we studied subcellular localization of endogenous PKD in BV-2 cells and PMM in response to LPA. In unstimulated BV-2 cells, tubulin and PKD2 was detected mainly at perinuclear areas (Fig. 3a, upper panel). After LPA stimulation (Fig. 3a, lower panel), cell area increased and induced the formation of new tubulin-positive plasma membrane protrusions, likely lamellipodia. PKD2 translocated to newly formed tubulin-positive membrane projections (Fig. 3a, lower panel).

In untreated PMM, PKD1 shows nuclear and cytosolic (in cellular protrusions/extensions) staining (Fig. 3b, upper panel). In response to LPA, PMM acquired a flattened morphology (tomato lectin staining) and a major part of originally cytosolic PKD1 translocated to the nucleus (Fig. 3b, lower panel). In contrast, PKD2 translocation in response to LPA is less pronounced in PMM (Fig. 3c). No nuclear PKD2 staining was observed in untreated or LPA-treated cells. In the absence of LPA, the majority of PKD2 was detected in membrane protrusions and as diffuse staining in the cytosol (Fig. 3c, upper panel). In response to LPA, PKD2 was (as in untreated cells) still detected in the cytosol (Fig. 3c, lower panel).

### The LPAR5/PKD axis controls the activation of pro-inflammatory transcription factors

**Fig. 4:**
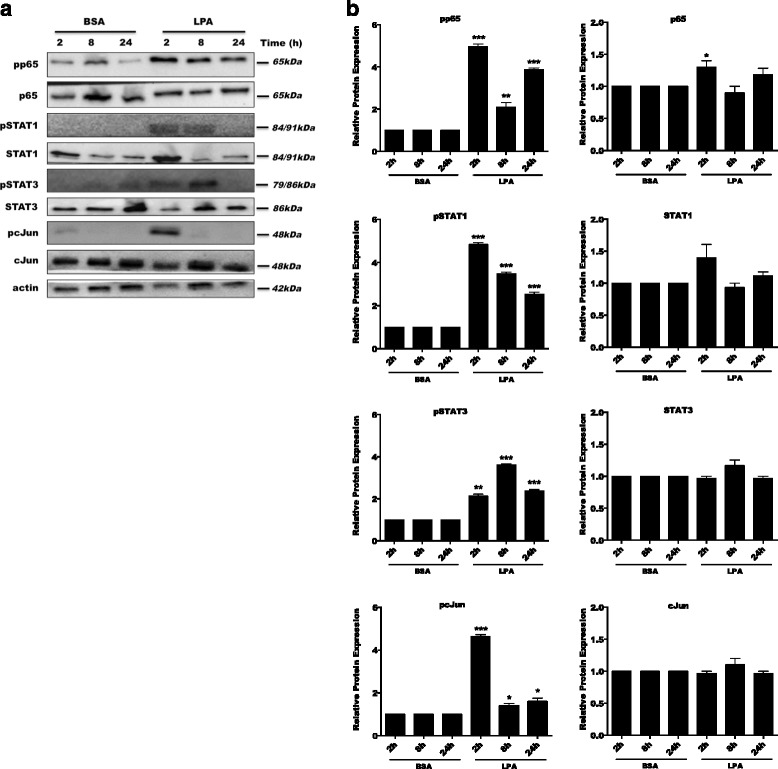
LPA induces the activation of pro-inflammatory transcription factors. Serum-starved **a** PMM were treated with 0.1% BSA (control) or LPA (1 μM) for the indicated time periods, and cellular protein lysates were collected. Phosphorylation of p65-NF-κB, STAT1, STAT3, and c-Jun was analyzed by western blotting. One representative blot is shown (*N* = 3). Molecular mass is indicated at the right. Actin was used as loading control. **b** Densitometric analysis of western blots (*N* = 3). Results show the significance of changes in the protein expression and represent mean values + SEM (*p < 0.05, **p < 0.01, ***p < 0.001; unpaired Student’s *t* test; BSA versus LPA for each time point)

**Fig. 5:**
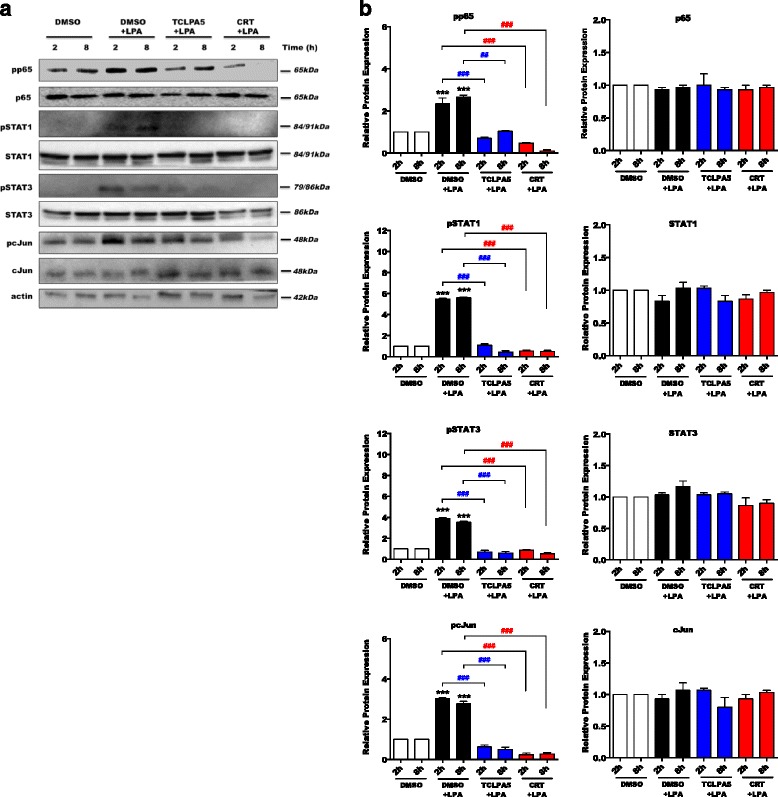
The LPAR5/PKD axis controls the phosphorylation of pro-inflammatory transcription factors. **a** PMM were seeded on 12-well plates, serum-starved, and incubated with DMSO, DMSO plus LPA (1 μM), and LPA (1 μM) in the presence of TCLPA5 (5 μM) or CRT0066101 (1 μM) for the indicated time periods. The phosphorylation of p65-NF-κB, STAT1, STAT3, and c-Jun was detected by western blotting. One representative blot is shown (*N* = 3). **b** Densitometric analysis of western blots (*N* = 3). Results are presented as mean values + SEM (***p < 0.001, compared to DMSO-treated cells; ^##^ *p* < 0.01, ^###^ *p* < 0.001, LPA plus TCLPA5 or CRT versus DMSO + LPA; one-way ANOVA with the Bonferroni correction)

During earlier work, we unraveled LPAR5 as a critical receptor that controls an LPA-induced pro-inflammatory microglial phenotype [43]; here, we determined downstream signaling pathways that are activated by LPAR5. These experiments indicated that LPA induced phosphorylation of IKK, IκB, p65-NF-κB, STAT1, STAT3, and c-Jun in BV-2 cells (Additional file 3: Figure S3A). Pharmacological inhibition of LPAR5 and PKD1–3 by TCLPA5 and CRT0066101, respectively, attenuated phosphorylation of all transcription factors analyzed (Additional files 4 and 5: Figures S4 and S5). In primary microglia, phosphorylation of p65-NF-κB, STAT1, STAT3, and c-Jun was detectable after 2 or 8 h and gradually declined afterwards (Fig. 4a). Phosphorylation of p65-NF-κB remained upregulated until 24 h post LPA treatment. Inhibition of LPAR5 and PKD1–3 effectively mitigated transcription factor phosphorylation at both time points analyzed (Fig. 5).

### Transcriptional regulation of pro-migratory, pro-invasive, and pro-angiogenic factors after LPA treatment

**Fig. 6:**
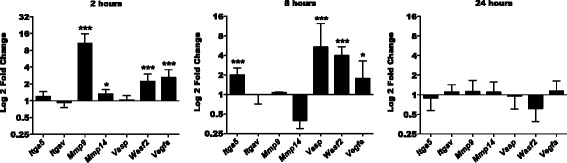
Effect of LPA treatment on pro-migratory, pro-invasive, and pro-angiogenic gene expression. PMM were seeded onto 24-well plates, serum-starved overnight, and treated with 0.1% BSA (control) or LPA (1 μM). At the indicated time points, RNA was isolated, reverse-transcribed, and analyzed by qPCR. Expression ratios were normalized to HPRT. Results of three separate experiments in triplicate are expressed as mean + SD (*p < 0.05, ***p < 0.001; REST; pairwise re-allocation test)

**Fig. 7:**
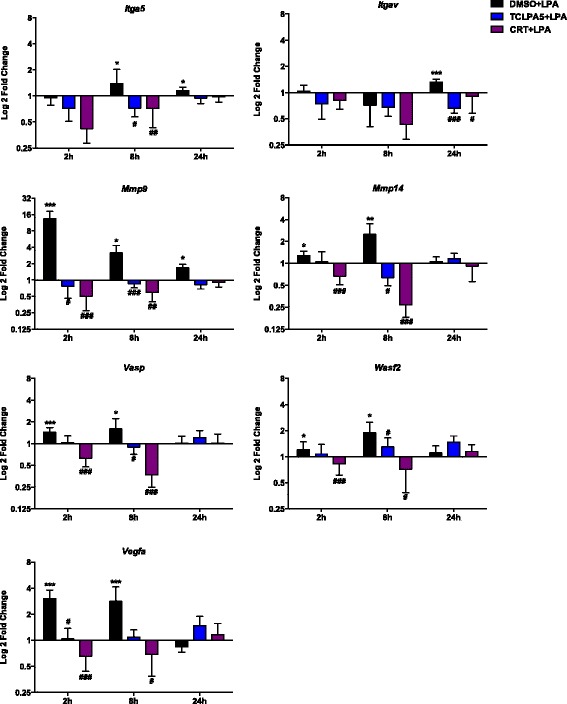
LPA controls the expression of pro-migratory, pro-invasive, and pro-angiogenic genes via the LPA/LPAR5/PKD axis. PMM were cultured in 24-well plates, serum-starved overnight, and treated with DMSO, DMSO plus LPA (1 μM), and LPA (1 μM) plus TCLPA5 (5 μM) or CRT (1 μM). At the indicated time points, cells were scraped, RNA was isolated and reverse-transcribed, and the gene products indicated were analyzed by qPCR. Expression ratios were normalized to HPRT expression. Results of three separate experiments performed in triplicate are expressed as mean + SD (*p < 0.05, **p < 0.01, ***p < 0.001, compared to vehicle control; ^#^ *p* < 0.05, ^##^ *p* < 0.01, ^###^ *p* < 0.001, cells treated with each inhibitor plus LPA compared to LPA-treated cells; REST; pairwise re-allocation test)

Following a chemotactic signal, microglia migrate towards the site of lesion and activate transcriptional programs that result in phenotypic transformation. We chose to analyze the effect of LPA on a selected set of migration/invasion-related genes in PMM in the absence or presence of the LPAR5/PKD inhibitors. Two hours post LPA (1 μM) treatment, *Mmp9*, *Mmp14*, *Wasf2*, and *Vegfa* gene expression was upregulated. At 8 h, *Itga5*, *Vasp*, *Wasf2*, and *Vegfa* were increased more than twofold and returned to or below baseline after 24 h (Fig. 6). Inhibitor studies revealed that both TCLPA5 and CRT0066101 reversed the effects of LPA on *Itga5*, *Itgav*, *Mmp9*, *Mmp14*, *Vasp*, *Wasf2*, and *Vegfa* expression (Fig. 7).

### LPA induces cytoskeletal and morphological rearrangements

**Fig. 8:**
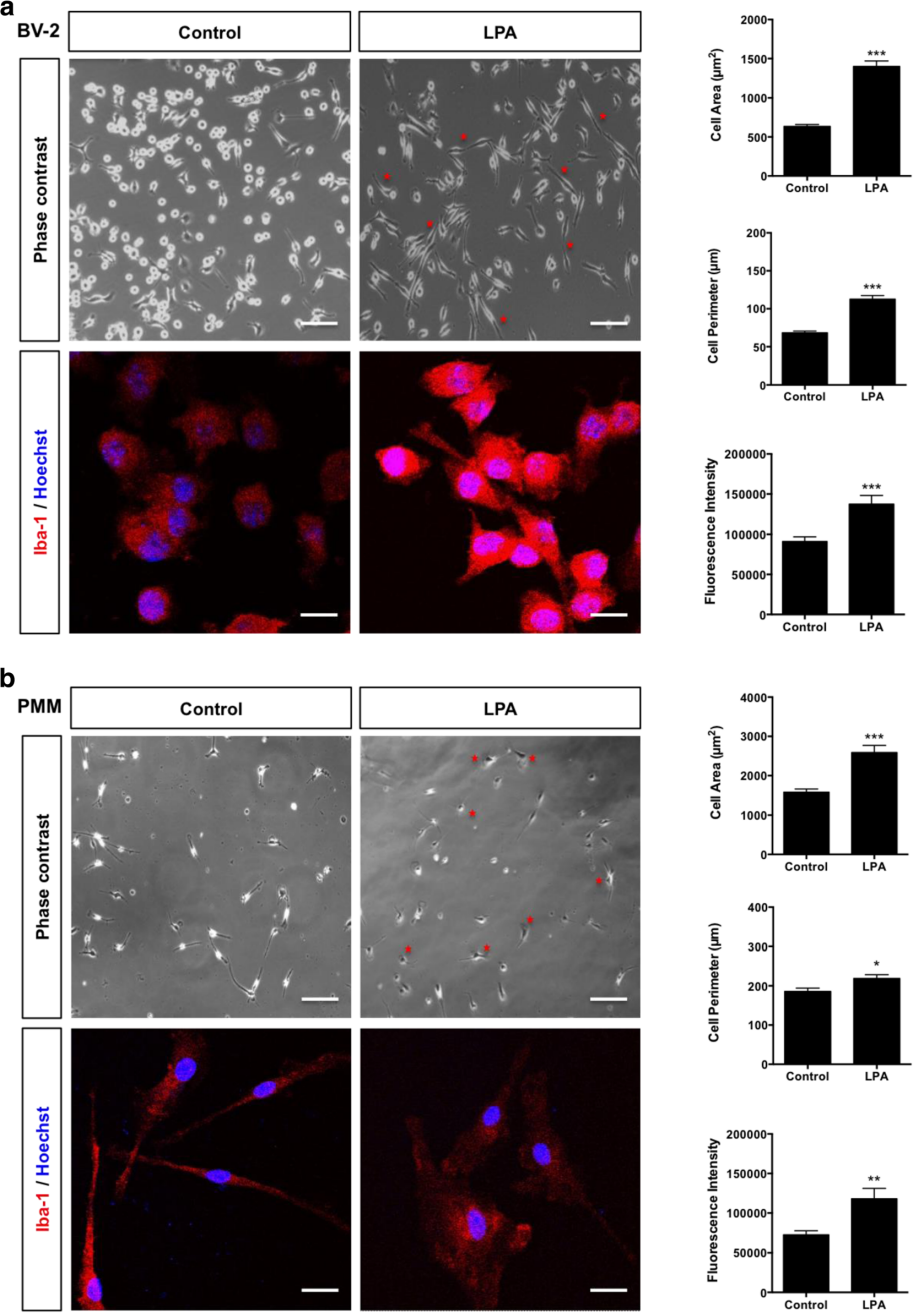
LPA alters BV-2 and PMM morphology. **a** BV-2 and **b** PMM were cultured on Permanox slides and serum-starved overnight prior LPA treatment (1 μM; 24 h). Cells were incubated with rabbit anti-mouse Iba-1 antibody (1:100) and Cy-3-labeled secondary goat anti-rabbit antibody (1:200), mounted, and examined by a Leica confocal microscope. Representative micrographs depict morphological changes upon LPA treatment. Morphological analysis (cell area and perimeter) and analysis of Iba-1 fluorescence intensity were performed using ImageJ (right panels). At least 50 cells out of three different areas per chamber were measured in two independent experiments. The results are presented as mean + SEM (*p < 0.05, **p < 0.01, ***p < 0.001; unpaired Student’s *t* test). Scale bars (phase contrast) = 200 μm; scale bars (Iba-1) = 20 μm

**Fig. 9:**
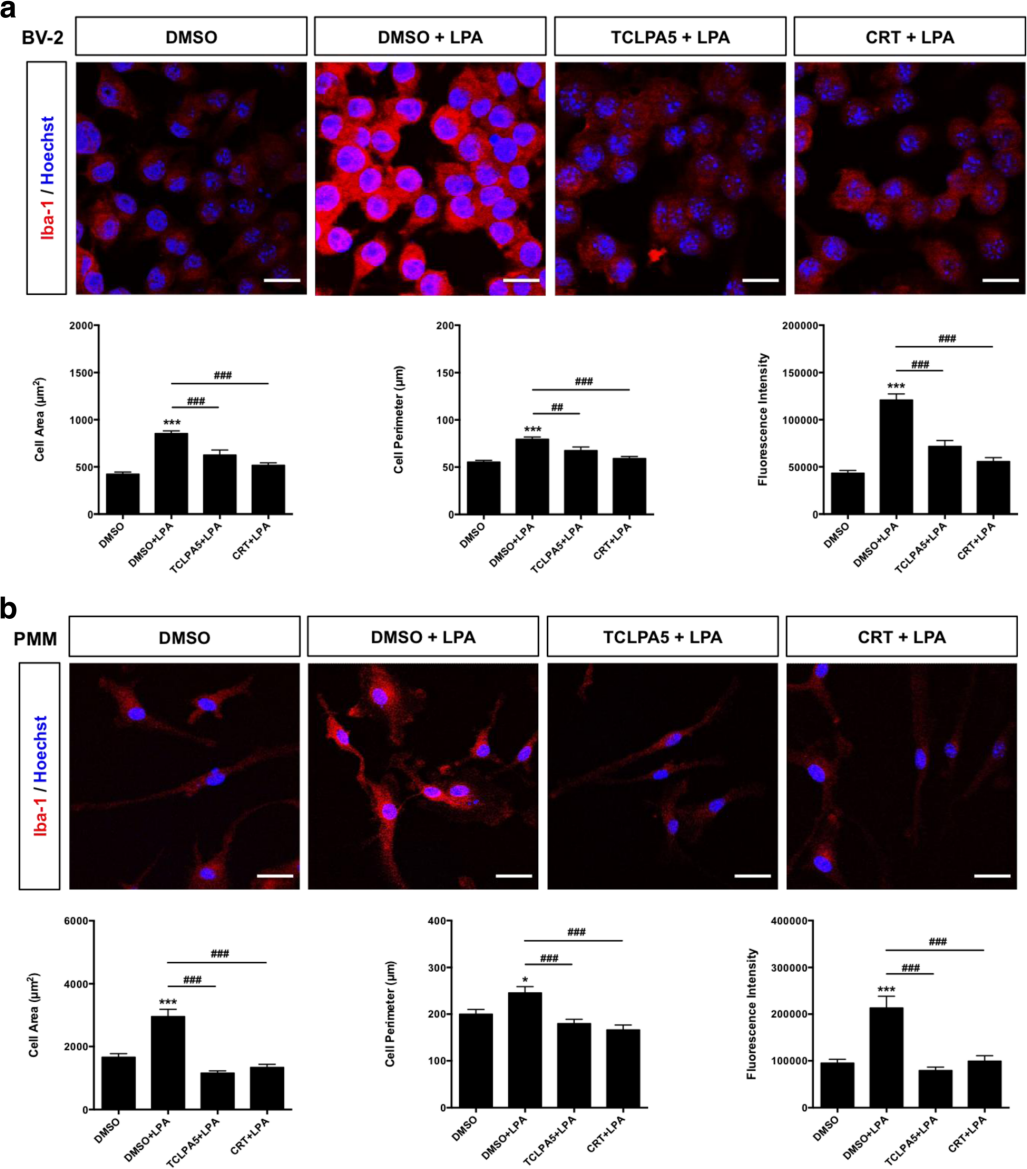
Inhibition of LPAR5 and PKD reverses the LPA-induced morphological changes in microglial cells. **a** BV-2 and **b** PMM were cultured on chamber slides, serum-starved overnight, and treated with DMSO, DMSO plus LPA (1 μM), and LPA (1 μM) plus TCLPA5 (5 μM) or CRT (1 μM) for 24 h. Cells were fixed, permeabilized, blocked, and incubated with rabbit anti-mouse Iba-1 antibody (1:100) and, subsequently, with Cy-3-labeled secondary goat anti-rabbit antibody (1:200). Images were acquired with a Leica confocal microscope. Morphological analysis and analysis of Iba-1 fluorescence intensity were performed using ImageJ. At least 50 cells out of three different areas per chamber were measured (two independent experiments), and quantitative results are shown as bar graphs below fluorescence images. The results are presented as mean + SEM (*p < 0.05, ***p < 0.001, compared to DMSO-treated cells; ^##^ *p* < 0.01, ^###^ *p* < 0.001, LPA plus TCLPA5 or CRT versus LPA only; unpaired Student’s *t* test). Scale bars = 20 μm

In the presence of LPA, BV-2 cells (Fig. 8a) and PMM (Fig. 8b) changed morphology. This transformation was associated with increased cell area, cell perimeter, and Iba-1 fluorescence intensity (bar graphs in Fig. 8). LPAR5 antagonism by TCLPA5 significantly attenuated LPA-mediated morphological alterations and Iba-1 fluorescence in both BV-2 and primary microglia (Fig. 9a, b). In line with biological functions in other cells, the pan PKD antagonist CRT0066101 repressed the morphological transformation of both cell types (Fig. 9a, b). These data indicate that LPAR5 acts as a sensor and at least one member of the PKD family acts as a transducer of LPA-mediated signals that impact microglial morphology and activation.

### The migratory response of microglia is under LPAR5 and PKD control

**Fig. 10:**
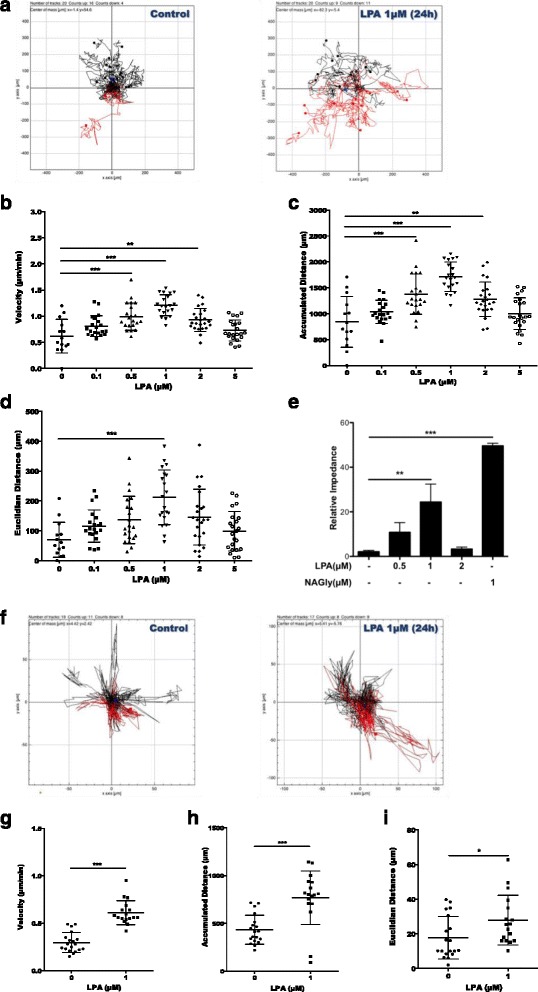
LPA induces microglial chemokinesis and chemotaxis. **a** BV-2 cells were cultured in 24-well plates, serum-starved overnight, and incubated with 0.1% BSA or with different LPA concentrations for 24 h. Migration was analyzed by time-lapse microscopy. **b** Velocity, **c** accumulated distance, and **d** Euclidean distance of at least 20 cells per sample were determined by ImageJ. **e** Chemotaxis was analyzed using the xCELLigence system. Serum-starved cells were allowed to migrate across uncoated Transwell inserts (CIM plates) for 24 h. Chemotaxis [0.1% BSA or LPA (1 μM) added to the lower compartment] was followed in real time by continuous electrical impedance measurement. NAGly (1 μM) was used as positive migration control. **f** PMM were cultured on PDL-coated 24-well plates, serum-starved overnight, and treated with 0.1% BSA or LPA (1 μM). Time-lapse microscopy was used to analyze 2D migration of at least 20 viable cells per sample per condition. **g** Velocity, **h** accumulated distance, and **i** Euclidean distance were determined using ImageJ. For BV-2 cells, results from three independent experiments performed in triplicate were expressed as mean + SD (**p < 0.01, ***p < 0.001; one-way ANOVA with the Bonferroni correction LPA-treated versus untreated). For PMM, the results from two experiments in triplicate are shown as mean + SEM (*p < 0.05, ***p < 0.001; unpaired Student’s *t* test)

Having established activation of signaling cascades that are potential drivers of cell locomotion and morphological transition, we examined the impact of LPA on microglial chemokinesis and chemotaxis. Using time-lapse video microscopy, we analyzed the tracks of individual control and LPA-treated BV-2 cells (Fig. 10a). The concentration-dependent migrational response of BV-2 cells to LPA (Fig. 10b) is bell-shaped, reaching a maximum at 1 μM and returning almost to baseline at 5 μM of LPA. The mean velocity of unstimulated cells was 0.6 μm/min and increased twofold in response to 1 μM LPA (Fig. 10b). The total accumulated migration distance increased twofold (Fig. 10c, control vs. 1 μM LPA), and the Euclidean distance increased threefold (Fig. 10d, control vs. 1 μM LPA).

Chemotaxis experiments were performed in real time using the xCELLigence system. Migration of serum-starved cells across uncoated CIM plates was studied in the absence or presence of LPA in the lower chamber of the Transwell inserts. NAGly that drives migration through GPR18 [59] was used as positive control. LPA induced directional migration at 0.5 and 1 μM LPA as compared to controls (Fig. 10e). LPA at 2 μM had no effect on directional migration. NAGly induced a twofold higher increase in migration as observed for 1 μM LPA (Fig. 10e). PMM also responded to LPA with increased chemokinesis. Tracks of primary cells studied in the absence (left panel) or presence (right panel) of LPA are shown in Fig. 10f. LPA treatment increased cell velocity (2-fold) as well as accumulated (1.8-fold) and Euclidean (1.5-fold) distances (Fig. 10g–i).

**Fig. 11:**
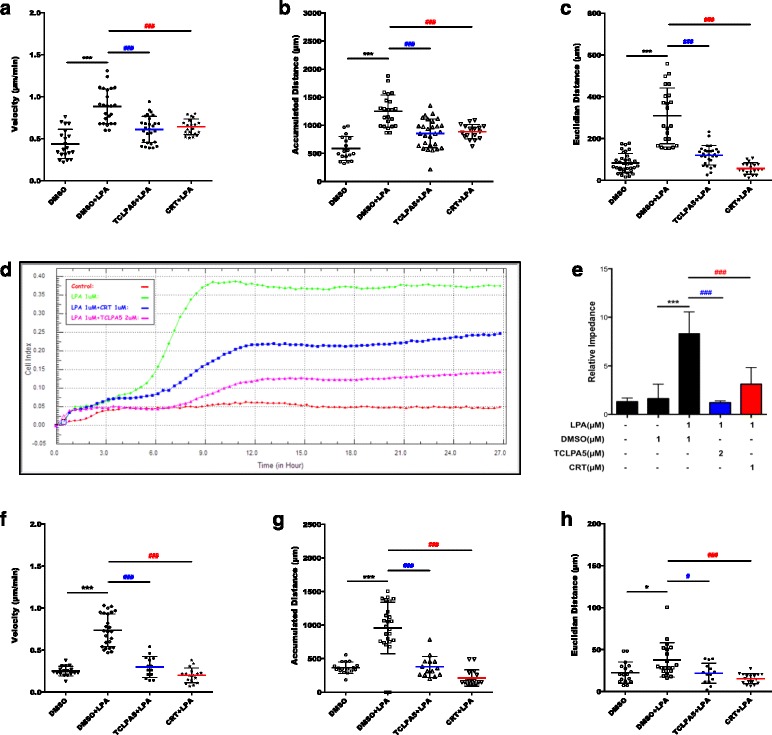
LPAR5 and PKD family control the chemotactic response of microglia. BV-2 microglial cells were cultured in a 24-well plate, serum-starved overnight, and treated with DMSO, DMSO plus LPA (1 μM), and LPA (1 μM) plus TCLPA5 (5 μM) or CRT (1 μM). **a** Velocity, **b** accumulated distance, and **c** Euclidean distance were determined using time-lapse microscopy. **d**, **e** Serum-starved BV-2 cells were incubated with LPA in the absence or presence of the indicated antagonists and allowed to migrate across Transwell inserts (CIM plates) for 24 h. Real-time cell migration was monitored using the xCELLigence system. **f**–**h** PMM were cultured in PDL-coated 24-well plates, serum-starved overnight, and treated with DMSO, DMSO plus LPA (1 μM), and LPA (1 μM) plus TCLPA5 (5 μM) or CRT (1 μM). **f** Velocity, **g** accumulated distance, and **h** Euclidean distance were analyzed by time-lapse microscopy. For BV-2 cells, results from three independent experiments performed in triplicate are presented as mean + SD (***p < 0.001, compared to DMSO; ^###^ *p* < 0.001, cells treated with the inhibitor plus LPA compared to LPA-treated cells; one-way ANOVA with the Bonferroni correction). For PMM, results from two experiments in triplicate are shown as mean + SEM (*p < 0.05, ***p < 0.001, compared to DMSO; ^#^ *p* < 0.05, ^###^ *p* < 0.001, cells treated with the inhibitor plus LPA compared to LPA-treated cells; one-way ANOVA with the Bonferroni correction)

Pharmacological antagonism of LPAR5 or PKD by TCLPA5 or CRT0066101 effectively attenuated chemokinesis of BV-2 cells (Fig. 11a–c). Chemotaxis experiments using BV-2 in the xCELLigence system (Fig. 11d) revealed that both inhibitors decreased migration to the lower chamber. Statistical evaluation of relative impedance values (24-h LPA treatment) is shown in Fig. 11e. Both TCLPA5 and CRT0066101 decreased chemokinetic parameters of PMM back to baseline values (Fig. 11f–h).

**Fig. 12:**
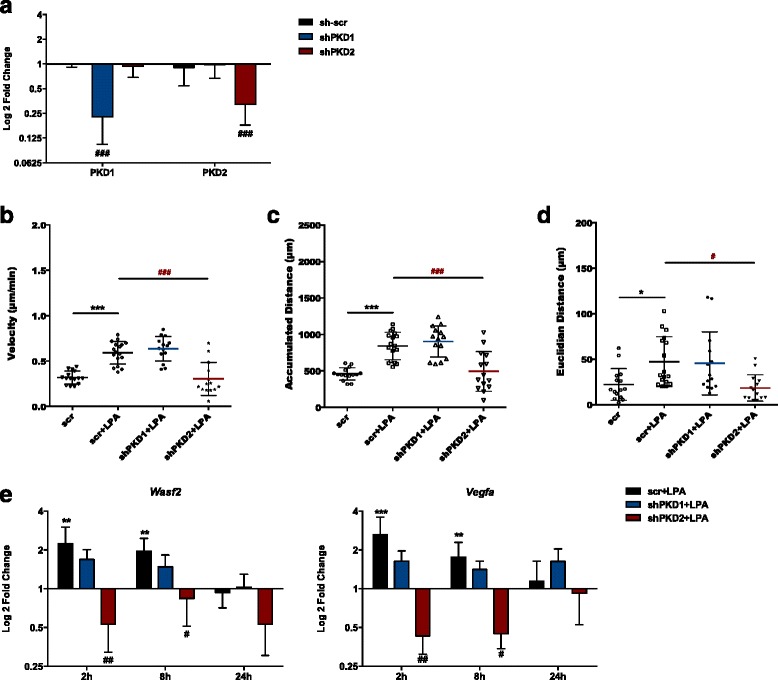
PKD2 is mainly responsible for a motile PMM phenotype. PMM were cultured in PDL-coated 24-well plates and transduced by shPKD1 and shPKD2. Control vectors expressing scrambled shRNA were used as control. **a** Silencing efficacy was assessed 72 h post transduction by qPCR. **b**–**d** Cells were serum-starved overnight and treated with 0.1% BSA or 1 μM LPA for 24 h. Velocity, accumulated distance, and Euclidean distance were analyzed. **e** Serum-starved PMM incubated with 0.1% BSA or 1 μM LPA were lysed, RNA was isolated and reverse-transcribed, and the gene products indicated were analyzed by qPCR. Expression ratios are normalized to HPRT expression. Results of three separate experiments in triplicate are presented as mean + SD (*p < 0.05, ***p < 0.001, compared to DMSO; ^#^ *p* < 0.05, ^##^ *p* < 0.01, ^###^ *p* < 0.001, cells treated with the inhibitor plus LPA compared to LPA-treated cells; one-way ANOVA with the Bonferroni correction)

To be able to differentiate between effects mediated by PKD1 and/or PKD2 on the migration of PMM, we chose an shRNA approach. Silencing of PKD1 and PKD2 was efficient (in comparison to PMM transduced with scrambled shRNA, levels were reduced by 80 and 60%, respectively), and no effect on the non-targeted isoform was observed (Fig. 12a). Stimulation of scr-shRNA-transduced cells with LPA resulted in increased chemokinesis (Fig. 12b–d). Silencing of PKD1 had no effect, while PKD2 silencing resulted in complete inhibition of chemokinesis. Next, we sought to examine the impact of PKD1/PKD2 silencing on the selected set of migration/invasion-related genes. We observed that only two (Wasf2 and Vegfa) out of the seven LPA-upregulated genes (Fig. 6) were differentially affected by PKD1 and PKD2 silencing: PKD1 silencing had no significant effects on Wasf2 and Vegfa expression, while PKD2 silencing reduced the expression of both genes to or below baseline levels (Fig. 12e).

### PKD inhibition blunts secretion of cytokines and chemokines

**Fig. 13:**
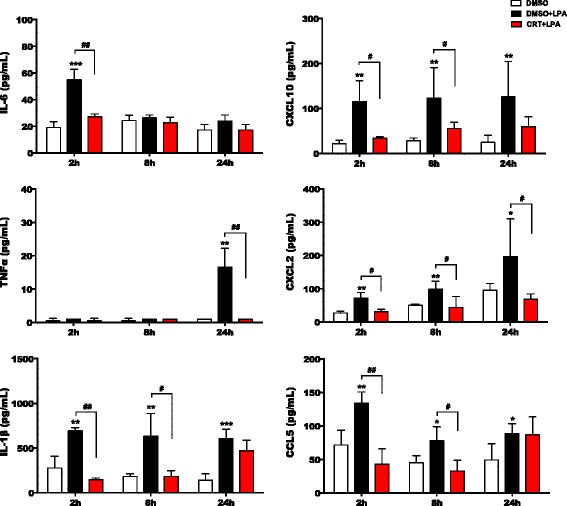
CRT0066101 abrogates the secretion of pro-inflammatory cytokines and chemokines. PMM were cultured on 24-well plates and serum-starved overnight. The supernatants were collected after incubation with DMSO, DMSO plus LPA (1 μM), or LPA (1 μM) plus CRT (1 μM). ELISAs were used to quantitate IL-6, IL-1β, CXCL10 (IP-10), TNF-α, CXCL2 (MIP-2), and CCL5 (RANTES) concentrations. Results shown represent mean + SD from three independent experiments (*N* = 3) performed in triplicate (*p < 0.05, **p < 0.01, ***p < 0.001, compared to vehicle control; ^#^ *p* < 0.05, ^##^ *p* < 0.01, CRT + LPA compared to LPA-treated cells; one-way ANOVA with the Bonferroni correction)

We have previously shown that LPA induces cytokine and chemokine secretion in microglia in an LPAR5-dependent manner [43]. Here, we show that IL-6, CXCL10, TNF-α, CXCL2, IL-1β, and CCL5 secretion was upregulated by LPA in BV-2 cells and significantly reduced by CRT0066101 (Additional file 6: Figure S6). Similar effects were observed in primary microglia (Fig. 13).

### PKD inhibition decreases LPA-induced expression of pro-inflammatory markers and microglial neurotoxicity

**Fig. 14:**
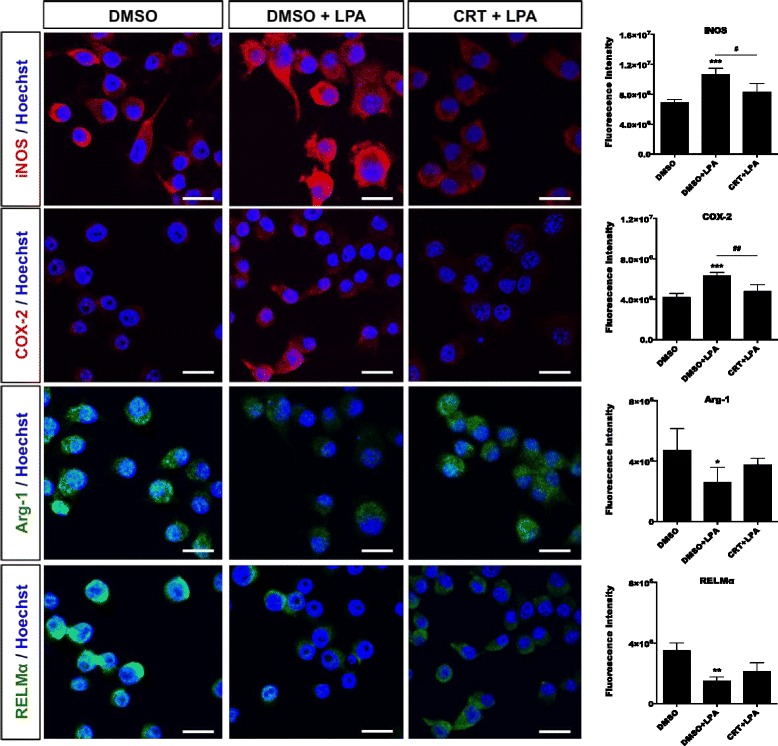
CRT0066101 reduces the expression of LPA-induced M1 markers. BV-2 cells were incubated in the presence of DMSO, DMSO plus LPA (1 μM), or LPA (1 μM) plus CRT (1 μM) for 24 h. Cells were stained for iNOS, COX-2, Arg-1, or RELMα and visualized using confocal microscopy. Fluorescence intensity was quantitated with ImageJ (bar graphs in right panels). At least 50 cells out of three different areas per chamber were measured. Results (three independent experiments) are presented as mean + SD (*p < 0.05, **p < 0.01, ***p < 0.001; ^#^ *p* < 0.05, ^##^ *p* < 0.01, inhibitor compared to LPA-treated cells; one-way ANOVA with the Bonferroni correction). Scale bars = 20 μm

**Fig. 15:**
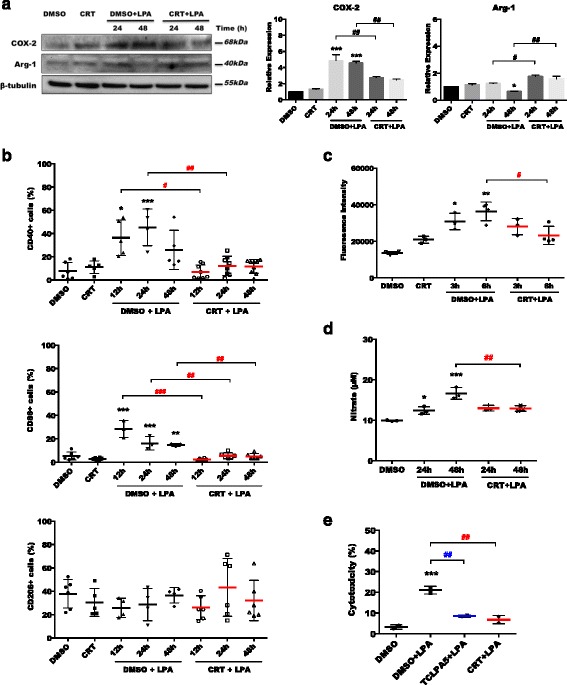
Inhibition of PKDs suppresses the LPA-induced pro-inflammatory phenotype. **a** Serum-starved BV-2 cells were treated with DMSO, DMSO plus LPA (1 μM), and LPA (1 μM) in the presence of CRT (1 μM) for 24 and 48 h. Cell lysates were collected, and expression of COX-2 and Arg-1 was analyzed by western blotting. One representative blot and the densitometric analysis (mean + SD) from three independent experiments are presented. **b** BV-2 cells were stained with PE-conjugated anti-CD40 (upper panel), APC-conjugated anti-CD86 (middle panel), or PE-conjugated anti-CD206 (lower panel) antibodies and analyzed using a Guava easyCyte 8 Millipore flow cytometer. Results from four independent experiments performed in triplicate are shown as mean value + SD. **c** Intracellular ROS levels generated in response to LPA by BV-2 cells were determined. Serum-starved cells were incubated with carboxy-H_2_DCFDA and treated with DMSO, DMSO plus LPA (1 μM), or LPA (1 μM) plus CRT (1 μM), and the fluorescence intensity was evaluated. Results (three independent experiments performed in triplicate) are presented as mean values + SD. **d** Serum-starved BV-2 cells were incubated with DMSO, DMSO plus LPA (1 μM), or LPA (1 μM) plus each inhibitor for the indicated times, and the production of NO was determined by measuring the total nitrate concentration in the supernatants. Data (two separate experiments performed in triplicate) are presented as mean values + SD. **e** CATH.a neurons were incubated for 24 h with conditioned media collected from DMSO or LPA-treated BV-2 cells cultured in the absence or presence of CRT (1 μM) for 24 h. LDH activity was determined in the neuronal supernatants after 24 h. Cytotoxicity was calculated according to the manufacturer’s instructions (*p < 0.05, **p < 0.01, ***p < 0.001, compared to DMSO-treated cells; ^#^ *p* < 0.05, ^##^ *p* < 0.01, ^###^ *p* < 0.001, each inhibitor compared to LPA-treated cells; one-way ANOVA with the Bonferroni correction)

We performed immunofluorescence staining and analysis using confocal microscopy (Fig. 14). These experiments revealed induction of iNOS and COX-2 in response to LPA (1 μM; 24 h), while the M2 markers Arg-1 and RELMα were decreased. Pretreatment with CRT0066101 attenuated iNOS and COX-2 expression whereas Arg-1 and RELMα levels were slightly increased (Fig. 14, bar graphs). Immunoblotting experiments supported the abovementioned results. Treatment with CRT0066101 reduced LPA-dependent COX-2 expression and increased expression of the M2 marker Arg-1 (Fig. 15a). Bar graphs represent densitometric evaluation of immunoreactive bands of three independent experiments (Fig. 15a, right panel). Using flow cytometry, we then analyzed the expression pattern of different surface markers in LPA-stimulated BV-2 cells in the absence or presence of CRT0066101. As shown in Fig. 15b, PKD inhibition abrogated LPA-induced CD40 and CD86 expression, whereas CD206 levels were unaffected at all time points investigated (Fig. 15b).

We further examined the impact of CRT0066101 on ROS and nitric oxide (NO) production in BV-2 cells. LPA increased ROS and NO concentrations, while both were significantly reduced by PKD antagonism (Fig. 15c, d). Finally, we determined potential neurotoxicity of the LPA-induced BV-2 secretome. CATH.a neurons were incubated with conditioned media collected from LPA-treated (in the absence or presence of TCLPA5 or CRT0066101) BV-2 cells. Neuronal cell death was quantified using an LDH activity kit. BV-2 medium collected from LPA-stimulated cells was cytotoxic for CATH.a neurons as evident from the fourfold increase in LDH activity (Fig. 15e). In contrast, the medium obtained from LPA-treated microglia in the presence of LPAR5 or PKD inhibitor did not affect neuronal viability.

## Discussion

Microglia are versatile players in both inflammatory and physiological conditions and must exhibit plasticity towards extracellular signals to be able to maintain CNS homeostasis. Microglial dysfunction and neuroinflammation are implicated in the initiation and progression of many neurological diseases [60–62]. LPA levels increase under inflammatory conditions and in response to brain injury [63–65] and can be manipulated via ATX inhibition or lipid phosphate phosphatase-mediated degradation [66, 67]. Prenatal exposure to elevated LPA levels and dysregulated LPA signaling may have chronic effects and can be prevented through inhibition of different LPARs [16, 68, 69]. LPA signaling drives diverse physiological and pathophysiological processes within the nervous system [69] and might play an important role as a mediator of microglial activation in response to CNS injury. Of note, a recent study describes a newly developed LPAR5 antagonist (AS2717638) that exhibits potent analgesic effects against neuropathic and inflammatory pain in rodent models [70]. In this context, our data identify a critical role for LPA-induced signaling events that provide a *go* signal via the LPA/LPAR5/PKD axis in microglia. Here, we present evidence that exogenous LPA alters signaling, transcription factor phosphorylation, morphology, locomotion, inflammatory response, and neurotoxicity of microglia via PKD-mediated signaling pathways. These findings demonstrate that the PKD pathway couples LPAR5 signaling to a motile and inflammatory microglial phenotype.

PKD isoforms are key mediators of stress signals and, as such, impact a variety of signaling pathways and cellular functions including actin remodeling, vesicle trafficking, and exocytosis, cell motility, survival, and gene transcription [71]. They are new players among the signaling proteins that control nervous system function and regulate neuropathic pain transmission, neuronal polarity, and associative learning in *Caenorhabditis elegans* [72]. PKD members are recruited to different subcellular compartments in response to activation [49, 73]. LPA treatment of BV-2 cells and PMM induced altered intracellular trafficking of PKD2 and/or PKD1. In BV-2 cells, LPA induced a pronounced relocation of PKD2 from perinuclear localization to newly formed membrane protrusions. Whether this is an indication for PKD-dependent actin remodeling as reported for PKD1 [74] is currently unclear. In LPA-treated primary microglia, PKD2 showed a perinuclear and cytoplasmic distribution. It has been demonstrated that the regulation of PKD2 trafficking is distinct from other PKD isoforms and PKD2 activation did not induce its redistribution from the cytoplasm to the nucleus [75]. In PMM, a major part of originally cytosolic PKD1 undergoes translocation to the nuclear compartment in response to LPA. This is in line with results reported for fibroblasts and epithelial cells, where cell stimulation with GPCR agonists resulted in nuclear accumulation of PKD1 that was prevented by inhibiting PKC activation [76].

Our results demonstrate that exogenous LPA induced activation of the PKD, MAPK, and AKT pathways, as well as phosphorylation of NF-κB, STAT1, STAT3, and c-Jun in a CRT0066101-sensitive manner both in BV-2 and PMM. All of these signaling pathways were reported to be involved in microglial polarization [77] and chemotaxis [78]. These findings indicate a sensor function for LPAR5 that transmits intracellular signals via PKD isoforms. This coincides with reports by other groups demonstrating ERK1/ERK2 activation via PKD-mediated phosphorylation of Ras and Rab interactor 1 [79]. Both p38 MAPK and JNK are also downstream targets of PKDs since PKD1 silencing attenuates p38 MAPK and JNK activation [80], and we have previously shown that AKT activation is under control of PKD2 [81]. In addition, PKD isoforms directly activate the NF-κB pathway: In HeLa cells, PKD1 activates NF-κB via oxidative stress signaling [82], while PKD2 supports the pIKKβ degradation pathway, and PKD3 is responsible for p65 phosphorylation of NF-κB in prostate cancer cells [83]. In mast cells, PKDs play a pivotal role in FcεRI-induced cytokine production through transcription factor activation including c-Jun [84]. Whether or not STAT activation can occur via PKD-mediated pathways is currently unclear. However, autocrine activation of the JAK/STAT pathway via cytokines that are secreted in response to LPA stimulation would be a plausible alternative explanation for our observations.

The observed morphological microglial responses in response to LPA are reminiscent of morphometric analyses of Iba-1-positive human microglia: primed gray and white matter microglia have an average twofold increase in the cell surface area as compared to their ramified counterparts [85]. We found that LPA induces chemokinetic and chemotactic microglial migration, reaching maximum values at 1 μM LPA. Higher concentrations could not induce a migratory response. It is possible that at higher concentrations, alternative signaling pathways are activated. It has been reported that at concentrations ≥ 3 μM, LPA led to increased [Ca^2+^]_*c*_ signals and metabolic activity in mouse microglial cells [86]. Microglial migration was blunted by CRT0066101. In PMM, PKD1 silencing had no effects on LPA-stimulated locomotion, while PKD2 silencing augmented this stimulatory effect. This is in line with the pro-migratory function of PKD2 in cancer [83, 87, 88] and endothelial cells [89], and the fact that lysoPC induces monocyte migration in a PKD2-dependent manner [90]. The role of PKD3 was experimentally not addressed during the present study. Generally, PKD enzymes, dependent on activity level and stimulus, are modulators of cell migration. In HeLa cells, decreased basal activity of PKD3 (resulting in decreased serine/threonine protein kinase PAK4 activity and cofilin hypo-phosphorylation), or increased activities of PKD2 and PKD3 (resulting in additional inhibition of protein phosphatase Slingshot homolog 1 SSH1L and cofilin hyperphosphorylation), directly inhibited cell migration [91].

Increased chemokinesis and chemotaxis were accompanied by LPA-induced upregulation of *Itga5*, *Itgav*, *Mmp9*, *Mmp14*, *Vasp*, *Wasf2*, and *Vegfa*. All of these gene products play important roles in cell morphology, adhesion, migration, invasion, and angiogenesis, and all of them are under control of PKD-dependent pathways in other cellular systems: PKD1 phosphorylates rabaptin-5 in fibroblasts, a posttranslational event controlling α5β1 and αvβ3 recycling, thereby regulating cell migration [92]. In cancer cells, PKD2 represents a core factor in the formation of a multiprotein complex that controls secretion of MMPs from the trans-Golgi network [93]. The actin-associated protein Vasp is phosphorylated by PKD1 to increase filopodia formation in HeLa cells [74]. In microglia, Vasp phosphorylation induces membrane ruffling and chemotaxis [94]. The Wasf2 complex regulates lamellipodia formation and is under regulation of PKD1-mediated pathways in the pancreas and breast cancer cells [95]. Vegfa expression/secretion by gastrointestinal tumor cells and Vegf-stimulated blood vessel formation is upregulated by PKD2 [44, 96].

PKD isoforms are regulators of classical [97, 98] and extracellular vesicle-based [99] secretory pathways. This became evident in PKD2^−/−^ lymphoblasts showing significantly reduced secretion of IL-2 in response to T cell antigen receptor triggering [97]. In addition, exosome secretion is enhanced in a PKD1-dependent manner in T lymphocytes and is impaired in PKD2-deficient lymphoblasts and PKD1/PKD3 knockout B cells [99]. A proteome study in PKD2^−/−^ cytotoxic T cells revealed that PKD2 phosphorylates a number of downstream targets that are regulators of intracellular protein and vesicle trafficking pathways [100]. In line with these reports, we observed reduced secretion of cytokines from CRT0066101-treated microglia. Since LPA stimulates microvesicle release [101] and microglial cell-derived microvesicle cargo contains (pro)IL-1β and other parts of the inflammasome [102], it will be intriguing to see whether the LPA/LPAR5/PKD axis is involved in microvesicle shedding during a neuroinflammatory response.

Microglial cell-induced neurotoxicity [103] may be mediated by the constant increased production of pro-inflammatory cytokines and chemokines, NO [104], and ROS [105]. iNOS is not expressed in the healthy brain, but expression is induced in response to inflammatory mediators like LPS or cytokines. In microglia, upregulation of iNOS is proposed to be the leading source of NO production [106]. In response to iNOS upregulation, excess NO reacts with NADPH oxidase-derived O_2_^−^. This reaction results in the formation of the highly neurotoxic mediator peroxynitrite (ONOO^−^) in BV-2 microglia [107]. We observed increased NO and ROS production in response to LPA treatment that was reduced in the presence of CRT0066101. Increased expression of these oxidative stressors has been suggested to have deleterious effects (e.g., cell membrane damage, lipid denaturation, changes in the inner proteins, diminished antioxidant capacity of neurons) and promote the pathogenesis of many diseases [105, 108]. Here, we observed that supernatants that were collected from LPA-treated BV-2 cells induced cytotoxicity towards CATH.a neurons that was blocked by CRT0066101. This is in line with decreased cytotoxicity of PKD2^−/−^ T lymphoblasts [99].

Emerging evidence supports the fact that glioblastoma cells exert a significant influence on microglia/macrophages to hijack their antitumor functions [109, 110] and to develop strategies that facilitate a *hostile takeover* of these cells (reviewed in [111]). Interestingly, in glioblastoma multiforme (GBM) (an incurable brain cancer entity [112]), ATX is overexpressed [113], and some of our findings in LPA-stimulated cells are reminiscent of what was reported for glioma-associated microglia [110, 114]. As observed, COX-2 (Ptgs2), IL-1β, and CCL5 are among the 25 highest upregulated genes in glioma-associated microglia/macrophages [114]. Upregulated IL-6 production in microglia stimulates glioma invasiveness, and it was suggested that the CCL2/CCR2/IL-6 loop represents a potential target to interfere with glioma invasion [115]. Glioma-derived versican converts microglia into a pro-tumorigenic phenotype characterized by the upregulation of MMP9 and MMP14 (as observed here in response to LPA); in particular, MMP14 promotes activation of GBM-derived MMP2 that favors the invasive potential of malign glioblastoma cells [110]. Considering that interference with PKD activity inhibits GBM growth in vitro and in vivo [81], this target could hold promise to interfere with GBM progression and reprogram the tumor microenvironment. This is substantiated by findings from the present study where CRT0066101 inhibited LPA-mediated secretion of pro-tumorigenic chemokines/cytokines, MMPs, and expression of pro-angiogenic Vegfa.

## Conclusions

**Fig. 16:**
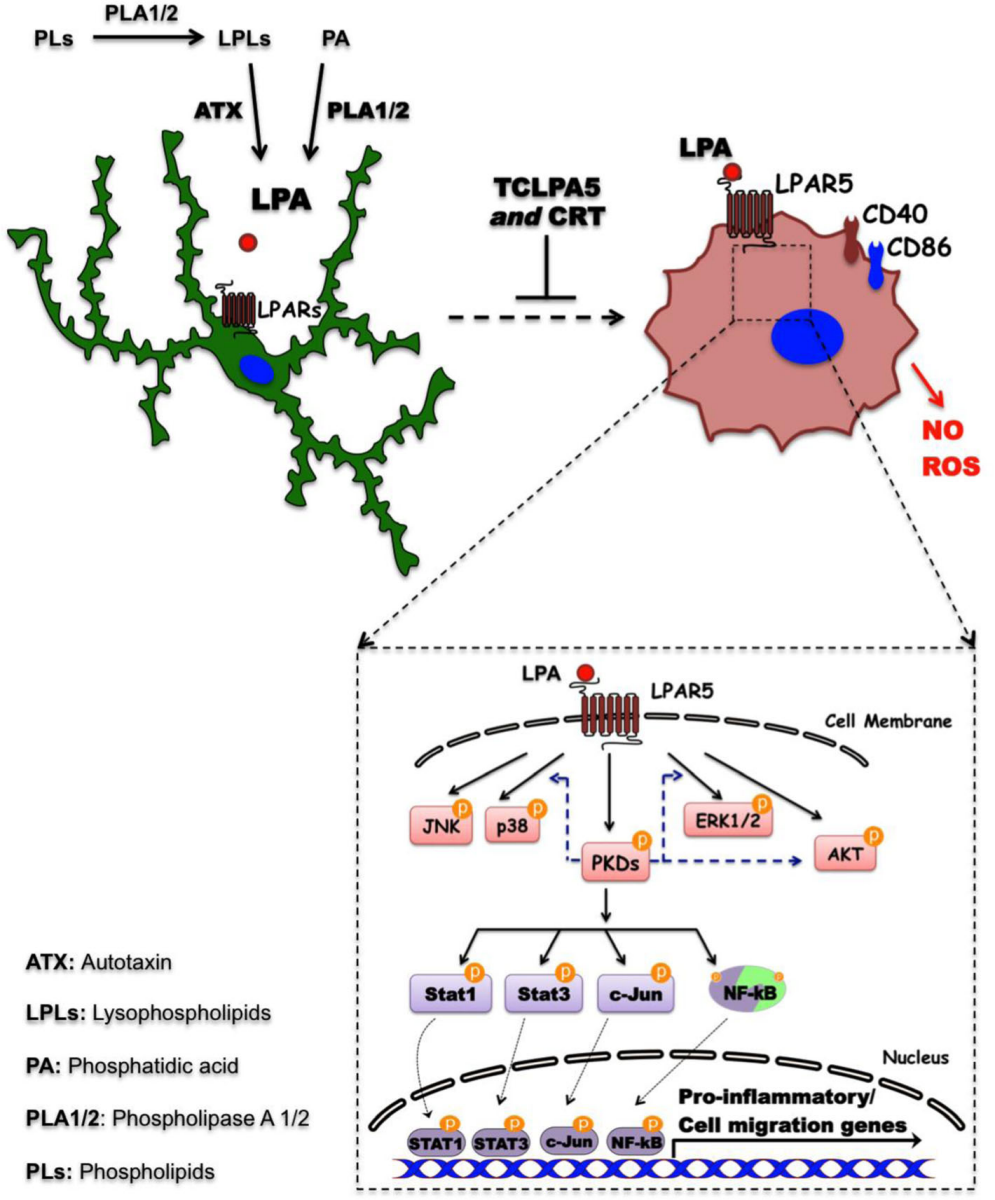
Graphical summary of findings obtained during this study. Inflammatory levels of LPA increased the migratory response of microglia and promoted pro-inflammatory signaling via the LPAR5/PKD axis. Interference with this signaling axis reduced microglial migration, blunted microglial cytotoxicity, and abrogated the expression and secretion of pro-inflammatory mediators (the steps between LPAR5 and PKDs, MAPKs, and AKT activation are not shown since those parts of the pathways were not experimentally addressed during the present study)

In the present study, we show that inflammatory LPA levels increased the migratory response of microglia and promoted a pro-inflammatory phenotype. Here, we demonstrate that this phenotype is induced via the LPAR5/PKD axis. Interference with this signaling cascade (using the pharmacological antagonists TCLPA5 and CRT0066101 or shRNA specific for PKD2) reduced microglial migration, blunted microglial cytotoxicity, and abrogated the expression and secretion of pro-inflammatory mediators. Our results (graphically summarized in Fig. 16) are a critical step towards a better understanding of LPA-mediated effects on the immune cells of the CNS and can foster the study of LPA signaling and its impact on microglial function in the diseased brain. Interference with various members of the LPA pathway, depending on the context of disease, may unravel potentially new targets to modulate neuroinflammation that have so far not been considered.

## Additional files

Additional file 1: Figure S1. LPAR5 controls LPA-mediated downstream signaling. (**A**) BV-2 microglia cells were cultured in 6-well plates and serum-starved overnight. The cells were preincubated with TCLPA5 (5 μM) for the indicated times and then incubated with LPA (1 μM) or LPA (1 μM) plus TCLPA5. Cells incubated only with 0.1% BSA or TCLPA5 (5 μM) were used as negative control. The phosphorylation states of PKDs, JNK, AKT, ERK1/2, and p38 were detected using western blotting. One representative blot is shown. (**B**) Densitometric analysis of western blots (*N* = 3). Results represent mean values + SEM (***p < 0.001 compared to control; ^##^ *p* < 0.01, ^###^ *p* < 0.001 LPA plus TCLPA5 versus LPA; one-way ANOVA with Bonferroni correction). (PPT 846 kb)

Additional file 2: Figure S2. PKD family inhibition abrogates LPA-mediated downstream signaling. (**A**) BV-2 microglia cells were cultured in 6-well plates, serum-starved overnight and preincubated with CRT0066101 (‘CRT’, 1 μM) for the indicated time periods before incubation with LPA (1 μM) or LPA (1 μM) plus CRT. Cells incubated only with 0.1% BSA or CRT (1 μM) were used as negative control. The phosphorylation states of PKDs, JNK, AKT, ERK1/2, and p38 were detected by immunoblotting and one representative blot for each protein is shown. (**B**) Densitometric analysis of western blots (N = 3). Results are presented as mean values + SEM (***p < 0.001; compared to control; ^##^ *p* < 0.01, ^###^ *p* < 0.001 LPA plus CRT versus LPA; one-way ANOVA with Bonferroni correction). (PPT 777 kb)

Additional file 3: Figure S3. LPA promotes activation of pro-inflammatory transcription factors in BV-2 cells. Serum-starved (**A**) BV-2 cells were treated with 0.1% BSA (control) or LPA (1 μM) for the indicated time periods, the cellular protein lysates were collected and phosphorylation state of IKKα/β, IκBα, p65-NF-κB, STAT1, STAT3, and c-Jun was detected using immunoblotting. One representative blot out of N = 3 experiments is shown. Actin was used as loading control. (**B**) Densitometric analysis of western blots show the significance of changes in the protein expression and represent mean values + SEM (*p < 0.05, **p < 0.01, ***p < 0.001; one-way ANOVA with Bonferroni correction). (PPT 582 kb)

Additional file 4: Figure S4. TCLPA5 inhibits the phosphorylation of pro-inflammatory transcription factors. BV-2 microglia cells were cultured in 6-well plates and serum-starved overnight. Cells were treated with LPA (1 μM) or LPA (1 μM) in the presence of (**A**) TCLPA5 (5 μM) for the indicated time periods. Cells incubated only with 0.1% BSA or TCLPA5 (5 μM) were used as negative control. The phosphorylation of p65-NF-κB, STAT1, STAT3, and c-Jun was detected using western blotting and one representative blot is shown. (**B**) Densitometric analysis of western blots (N = 3). Results represent mean values + SEM (*p < 0.05, ***p < 0.001 compared to control; ^##^ *p* < 0.01, ^###^ *p* < 0.001 LPA plus TCLPA5 versus LPA; one-way ANOVA with Bonferroni correction). (PPT 511 kb)

Additional file 5: Figure S5. The phosphorylation of pro-inflammatory transcription factors is under PKD family control. BV-2 cells, serum-starved overnight and treated with LPA (1 μM) or LPA (1 μM) in the presence of (**A**) CRT0066101 (1 μM) for the indicated time periods. Cells incubated only with 0.1% BSA or CRT (1 μM) were used as negative control. The phosphorylation state of p65-NF-κB, STAT1, STAT3, and c-Jun was detected by western blotting. One representative blot is shown. (**B**) Densitometric analysis of western blots (N = 3). Results are presented as mean values + SEM (**p < 0.01, ***p < 0.001 compared to control; ^##^ *p* < 0.01, ^###^ *p* < 0.001 LPA plus CRT versus LPA; one-way ANOVA with Bonferroni correction). (PPT 500 kb)

Additional file 6: Figure S6. PKD family members control the secretion of pro-inflammatory cytokines and chemokines. BV-2 cells were cultured on 12-well plates; serum-starved o/n and the supernatants were collected after incubation with DMSO, DMSO plus LPA (1 μM) or LPA (1 μM) plus CRT (1 μM). ELISAs were used to quantitate IL-6, IL-1β, CXCL10 (IP-10), TNF-α, CXCL2 (MIP-2), and CCL5 (RANTES) concentrations. Results shown represent mean + SD from three independent experiments performed in triplicate (*p < 0.05; **p < 0.01 compared to vehicle control; ^#^ *p* < 0.05, ^##^ *p* < 0.01; CRT + LPA compared to LPA treated cells; one-way ANOVA with Bonferroni correction). (PPT 174 kb)

## References

[1] Prinz M, Mildner A (2011). Microglia in the CNS: immigrants from another world. Glia.

[2] Kettenmann H, Kirchhoff F, Verkhratsky A (2013). Microglia: new roles for the synaptic stripper. Neuron.

[3] Nimmerjahn A, Kirchhoff F, Helmchen F (2005). Resting microglial cells are highly dynamic surveillants of brain parenchyma in vivo. Science.

[4] Hirbec HE, Noristani HN, Perrin FE. Microglia responses in acute and chronic neurological diseases: what microglia-specific transcriptomic studies taught (and did not teach) us. Front Aging Neurosci. 2017;9(227) doi: 10.3389/fnagi.2017.00227. 10.3389/fnagi.2017.00227 PMC5519576 28785215

[5] Salter MW, Stevens B (2017). Microglia emerge as central players in brain disease. Nat Med.

[6] Lucin KM, Wyss-Coray T (2009). Immune activation in brain aging and neurodegeneration: too much or too little?. Neuron.

[7] Du L, Zhang Y, Chen Y, Zhu J, Yang Y, Zhang H-L. Role of microglia in neurological disorders and their potentials as a therapeutic target. Mol Neurobiol. 2016; doi: 10.1007/s12035-016-0245-0. 10.1007/s12035-016-0245-0 27830532

[8] Kierdorf K, Prinz M (2013). Factors regulating microglia activation. Front Cell Neurosci.

[9] Ransohoff RM, Perry VH (2009). Microglial physiology: unique stimuli, specialized responses. Annu Rev Immunol.

[10] Harry GJ, Kraft AD (2008). Neuroinflammation and microglia: considerations and approaches for neurotoxicity assessment. Expert Opin Drug Metab Toxicol.

[11] Ransohoff RM (2016). A polarizing question: do M1 and M2 microglia exist?. Nat Neurosci.

[12] Paolicelli RC, Jawaid A, Henstridge CM, Valeri A, Merlini M, Robinson JL, et al. TDP-43 depletion in microglia promotes amyloid clearance but also induces synapse loss. Neuron. 95(2):297–308.e6. doi: 10.1016/j.neuron.2017.05.037. 10.1016/j.neuron.2017.05.037 PMC5519492 28669544

[13] Keren-Shaul H, Spinrad A, Weiner A, Matcovitch-Natan O, Dvir-Szternfeld R, Ulland TK, et al. A unique microglia type associated with restricting development of alzheimer’s disease. Cell. 169(7):1276–90.e17. doi: 10.1016/j.cell.2017.05.018. 10.1016/j.cell.2017.05.018 28602351

[14] Krasemann S, Madore C, Cialic R, Baufeld C, Calcagno N, El Fatimy R, et al. The TREM2-APOE pathway drives the transcriptional phenotype of dysfunctional microglia in neurodegenerative diseases. Immunity. 47(3):566–81.e9. doi: 10.1016/j.immuni.2017.08.008. 10.1016/j.immuni.2017.08.008 PMC5719893 28930663

[15] Kihara Y, Mizuno H, Chun J (2015). Lysophospholipid receptors in drug discovery. Exp Cell Res.

[16] Yung YC, Stoddard NC, Chun J (2014). LPA receptor signaling: pharmacology, physiology, and pathophysiology. J Lipid Res.

[17] Choi JW, Chun J (2013). Lysophospholipids and their receptors in the central nervous system. Biochim Biophys Acta.

[18] Schulze C, Smales C, Rubin LL, Staddon JM (1997). Lysophosphatidic acid increases tight junction permeability in cultured brain endothelial cells. J Neurochem.

[19] On NH, Savant S, Toews M, Miller DW (2013). Rapid and reversible enhancement of blood-brain barrier permeability using lysophosphatidic acid. J Cereb Blood Flow Metab.

[20] Fukushima N, Weiner JA, Kaushal D, Contos JJ, Rehen SK, Kingsbury MA (2002). Lysophosphatidic acid influences the morphology and motility of young, postmitotic cortical neurons. Mol Cell Neurosci.

[21] Contos JJ, Fukushima N, Weiner JA, Kaushal D, Chun J (2000). Requirement for the lpA1 lysophosphatidic acid receptor gene in normal suckling behavior. Proc Natl Acad Sci U S A.

[22] Yung YC, Mutoh T, Lin ME, Noguchi K, Rivera RR, Choi JW (2011). Lysophosphatidic acid signaling may initiate fetal hydrocephalus. Sci Transl Med.

[23] Yu N, Lariosa-Willingham KD, Lin F-F, Webb M, Rao TS (2004). Characterization of lysophosphatidic acid and sphingosine-1-phosphate-mediated signal transduction in rat cortical oligodendrocytes. Glia.

[24] Pébay A, Torrens Y, Toutant M, Cordier J, Glowinski J, Tencé M (1999). Pleiotropic effects of lysophosphatidic acid on striatal astrocytes. Glia.

[25] Anliker B, Choi JW, Lin M-E, Gardell SE, Rivera RR, Kennedy G (2013). Lysophosphatidic acid (LPA) and its receptor, LPA1, influence embryonic schwann cell migration, myelination, and cell-to-axon segregation. Glia.

[26] Moller T, Contos JJ, Musante DB, Chun J, Ransom BR (2001). Expression and function of lysophosphatidic acid receptors in cultured rodent microglial cells. J Biol Chem.

[27] Bernhart E, Kollroser M, Rechberger G, Reicher H, Heinemann A, Schratl P (2010). Lysophosphatidic acid receptor activation affects the C13NJ microglia cell line proteome leading to alterations in glycolysis, motility, and cytoskeletal architecture. Proteomics.

[28] Schilling T, Repp H, Richter H, Koschinski A, Heinemann U, Dreyer F (2002). Lysophospholipids induce membrane hyperpolarization in microglia by activation of IKCa1 Ca(2+)-dependent K(+) channels. Neuroscience.

[29] Schilling T, Stock C, Schwab A, Eder C (2004). Functional importance of Ca2+-activated K+ channels for lysophosphatidic acid-induced microglial migration. Eur J Neurosci.

[30] Awada R, Rondeau P, Gres S, Saulnier-Blache JS, Lefebvre d’Hellencourt C, Bourdon E (2012). Autotaxin protects microglial cells against oxidative stress. Free Radic Biol Med.

[31] Sun L, Wu Z, Hayashi Y, Peters C, Tsuda M, Inoue K (2012). Microglial cathepsin B contributes to the initiation of peripheral inflammation-induced chronic pain. J Neurosci.

[32] Awada R, Saulnier-Blache JS, Gres S, Bourdon E, Rondeau P, Parimisetty A (2014). Autotaxin downregulates LPS-induced microglia activation and pro-inflammatory cytokines production. J Cell Biochem.

[33] Savaskan NE, Rocha L, Kotter MR, Baer A, Lubec G, van Meeteren LA (2007). Autotaxin (NPP-2) in the brain: cell type-specific expression and regulation during development and after neurotrauma. Cell Mol Life Sci.

[34] Tigyi G, Hong L, Yakubu M, Parfenova H, Shibata M, Leffler CW (1995). Lysophosphatidic acid alters cerebrovascular reactivity in piglets. Am J Phys.

[35] Ma L, Uchida H, Nagai J, Inoue M, Aoki J, Ueda H (2010). Evidence for de novo synthesis of lysophosphatidic acid in the spinal cord through phospholipase A2 and autotaxin in nerve injury-induced neuropathic pain. J Pharmacol Exp Ther.

[36] Santos-Nogueira E, Lopez-Serrano C, Hernandez J, Lago N, Astudillo AM, Balsinde J (2015). Activation of lysophosphatidic acid receptor type 1 contributes to pathophysiology of spinal cord injury. J Neurosci.

[37] Crack PJ, Zhang M, Morganti-Kossmann MC, Morris AJ, Wojciak JM, Fleming JK (2014). Anti-lysophosphatidic acid antibodies improve traumatic brain injury outcomes. J Neuroinflammation.

[38] Ueda H, Matsunaga H, Olaposi OI, Nagai J (2013). Lysophosphatidic acid: chemical signature of neuropathic pain. Biochim Biophys Acta.

[39] Inoue M, Rashid MH, Fujita R, Contos JJ, Chun J, Ueda H (2004). Initiation of neuropathic pain requires lysophosphatidic acid receptor signaling. Nat Med.

[40] Lin ME, Rivera RR, Chun J (2012). Targeted deletion of LPA5 identifies novel roles for lysophosphatidic acid signaling in development of neuropathic pain. J Biol Chem.

[41] Ma L, Uchida H, Nagai J, Inoue M, Chun J, Aoki J (2009). Lysophosphatidic acid-3 receptor-mediated feed-forward production of lysophosphatidic acid: an initiator of nerve injury-induced neuropathic pain. Mol Pain.

[42] Ma L, Nagai J, Ueda H (2010). Microglial activation mediates de novo lysophosphatidic acid production in a model of neuropathic pain. J Neurochem.

[43] Plastira I, Bernhart E, Goeritzer M, Reicher H, Kumble VB, Kogelnik N (2016). 1-Oleyl-lysophosphatidic acid (LPA) promotes polarization of BV-2 and primary murine microglia towards an M1-like phenotype. J Neuroinflammation.

[44] Wille C, Seufferlein T, Eiseler T (2014). Protein kinase D family kinases: roads start to segregate. BioArchitecture.

[45] Prigozhina NL, Waterman-Storer CM (2004). Protein kinase D-mediated anterograde membrane trafficking is required for fibroblast motility. Curr Biol.

[46] Storz P (2009). Protein kinase D1: a novel regulator of actin-driven directed cell migration. Cell Cycle.

[47] Fu Y, Rubin CS (2011). Protein kinase D: coupling extracellular stimuli to the regulation of cell physiology. EMBO Rep.

[48] Sumara G, Formentini I, Collins S, Sumara I, Windak R, Bodenmiller B (2009). Regulation of PKD by the MAPK p38delta in insulin secretion and glucose homeostasis. Cell.

[49] Rozengurt E (2011). Protein kinase D signaling: multiple biological functions in health and disease. Physiology.

[50] Chiu TT, Leung WY, Moyer MP, Strieter RM, Rozengurt E (2007). Protein kinase D2 mediates lysophosphatidic acid-induced interleukin 8 production in nontransformed human colonic epithelial cells through NF-kappaB. Am J Physiol Cell Physiol.

[51] Storz P, Doppler H, Toker A (2004). Activation loop phosphorylation controls protein kinase D-dependent activation of nuclear factor kappaB. Mol Pharmacol.

[52] Storz P, Doppler H, Toker A (2004). Protein kinase Cdelta selectively regulates protein kinase D-dependent activation of NF-kappaB in oxidative stress signaling. Mol Cell Biol.

[53] Zhu H, Yang Y, Zhang H, Han Y, Li Y, Zhang Y (2008). Interaction between protein kinase D1 and transient receptor potential V1 in primary sensory neurons is involved in heat hypersensitivity. Pain.

[54] Dusaban SS, Purcell NH, Rockenstein E, Masliah E, Cho MK, Smrcka AV (2013). Phospholipase C epsilon links G protein-coupled receptor activation to inflammatory astrocytic responses. Proc Natl Acad Sci U S A.

[55] Kozian DH, Evers A, Florian P, Wonerow P, Joho S, Nazare M (2012). Selective non-lipid modulator of LPA5 activity in human platelets. Bioorg Med Chem Lett.

[56] Harikumar KB, Kunnumakkara AB, Ochi N, Tong Z, Deorukhkar A, Sung B (2010). A novel small-molecule inhibitor of protein kinase D blocks pancreatic cancer growth in vitro and in vivo. Mol Cancer Ther.

[57] Halliwell B, Whiteman M (2004). Measuring reactive species and oxidative damage in vivo and in cell culture: how should you do it and what do the results mean?. Br J Pharmacol.

[58] Rozengurt E (2011). Protein kinase D signaling: multiple biological functions in health and disease. Physiology (Bethesda).

[59] McHugh D, SS H, Rimmerman N, Juknat A, Vogel Z, Walker JM (2010). N-Arachidonoyl glycine, an abundant endogenous lipid, potently drives directed cellular migration through GPR18, the putative abnormal cannabidiol receptor. BMC Neurosci.

[60] Han J, Harris RA, Zhang X-M (2017). An updated assessment of microglia depletion: current concepts and future directions. Molecular Brain.

[61] Donat CK, Scott G, Gentleman SM, Sastre M. Microglial activation in traumatic brain injury. Front Aging Neurosci. 2017;9(208) doi: 10.3389/fnagi.2017.00208. 10.3389/fnagi.2017.00208 PMC5487478 28701948

[62] Wolf SA, Boddeke HWGM, Kettenmann H (2017). Microglia in physiology and disease. Annu Rev Physiol.

[63] Balood M, Zahednasab H, Siroos B, Mesbah-Namin SA, Torbati S, Harirchian MH. Elevated serum levels of lysophosphatidic acid in patients with multiple sclerosis. Hum Immunol. 2014;75(5):411–3. doi:10.1016/j.humimm.2014.02.021. Epub 2014 Feb 12. 10.1016/j.humimm.2014.02.021 24530753

[64] Liu S, Murph M, Panupinthu N, Mills GB (2009). ATX-LPA receptor axis in inflammation and cancer. Cell Cycle.

[65] Lin ME, Herr DR, Chun J (2010). Lysophosphatidic acid (LPA) receptors: signaling properties and disease relevance. Prostaglandins Other Lipid Mediat.

[66] Aaltonen N, Lehtonen M, Varonen K, Goterris GA, Laitinen JT (2012). Lipid phosphate phosphatase inhibitors locally amplify lysophosphatidic acid LPA1 receptor signalling in rat brain cryosections without affecting global LPA degradation. BMC Pharmacol.

[67] Morris AJ, Smyth SS (2014). Lipid phosphate phosphatases: more than one way to put the brakes on LPA signaling?. J Lipid Res.

[68] Mirendil H, Thomas EA, De Loera C, Okada K, Inomata Y, Chun J (2015). LPA signaling initiates schizophrenia-like brain and behavioral changes in a mouse model of prenatal brain hemorrhage. Transl Psychiatry.

[69] Yung Yun C, Stoddard Nicole C, Mirendil H, Chun J. Lysophosphatidic acid signaling in the nervous system. Neuron. 85(4):669–82. doi: 10.1016/j.neuron.2015.01.009. 10.1016/j.neuron.2015.01.009 PMC4400838 25695267

[70] Murai N, Hiyama H, Kiso T, Sekizawa T, Watabiki T, Oka H (2017). Analgesic effects of novel lysophosphatidic acid receptor 5 antagonist AS2717638 in rodents. Neuropharmacology.

[71] Wood BM, Bossuyt J (2017). Emergency spatiotemporal shift: the response of protein kinase D to stress signals in the cardiovascular system. Front Pharmacol.

[72] Li G, Wang Y. Protein kinase D: a new player among the signaling proteins that regulate functions in the nervous system. Neurosci Bull. 2014; doi: 10.1007/s12264-013-1403-2. 10.1007/s12264-013-1403-2 PMC5562605 24526660

[73] Rozengurt E, Rey O, Waldron RT (2005). Protein kinase D signaling. J Biol Chem.

[74] Doppler HR, Bastea LI, Lewis-Tuffin LJ, Anastasiadis PZ, Storz P (2013). Protein kinase D1-mediated phosphorylations regulate vasodilator-stimulated phosphoprotein (VASP) localization and cell migration. J Biol Chem.

[75] Rey O, Yuan J, Rozengurt E. Intracellular redistribution of protein kinase D2 in response to G-protein-coupled receptor agonists. Biochem Biophys Res Commun. 2003;302(4):817–24. doi:10.1016/S0006-291X(03)00269-9. 10.1016/s0006-291x(03)00269-9 12646243

[76] Rey O, Sinnett-Smith J, Zhukova E, Rozengurt E (2001). Regulated nucleocytoplasmic transport of protein kinase D in response to G protein-coupled receptor activation. J Biol Chem.

[77] Popiolek-Barczyk K, Mika J (2016). Targeting the microglial signaling pathways: new insights in the modulation of neuropathic pain. Curr Med Chem.

[78] Fan Y, Xie L, Chung CY (2017). Signaling pathways controlling microglia chemotaxis. Mol Cells.

[79] Wang Y, Waldron RT, Dhaka A, Patel A, Riley MM, Rozengurt E (2002). The RAS effector RIN1 directly competes with RAF and is regulated by 14-3-3 proteins. Mol Cell Biol.

[80] Song J, Li J, Qiao J, Jain S, Mark Evers B, Chung DH (2009). PKD prevents H2O2-induced apoptosis via NF-kappaB and p38 MAPK in RIE-1 cells. Biochem Biophys Res Commun.

[81] Bernhart E, Damm S, Heffeter P, Wintersperger A, Asslaber M, Frank S (2014). Silencing of protein kinase D2 induces glioma cell senescence via p53-dependent and -independent pathways. Neuro-Oncology.

[82] Storz P, Doppler H, Toker A (2005). Protein kinase D mediates mitochondrion-to-nucleus signaling and detoxification from mitochondrial reactive oxygen species. Mol Cell Biol.

[83] Zou Z, Zeng F, Xu W, Wang C, Ke Z, Wang QJ (2012). PKD2 and PKD3 promote prostate cancer cell invasion by modulating NF-kappaB- and HDAC1-mediated expression and activation of uPA. J Cell Sci.

[84] Yamashita K, Gon Y, Shimokawa T, Nunomura S, Endo D, Miyata N (2010). High affinity receptor for IgE stimulation activates protein kinase D augmenting activator protein-1 activity for cytokine producing in mast cells. Int Immunopharmacol.

[85] Torres-Platas SG, Comeau S, Rachalski A, Bo GD, Cruceanu C, Turecki G (2014). Morphometric characterization of microglial phenotypes in human cerebral cortex. J Neuroinflammation.

[86] Möller T, Contos JJ, Musante DB, Chun J, Ransom BR (2001). Expression and function of lysophosphatidic acid receptors in cultured rodent microglial cells. J Biol Chem.

[87] Alpsoy A, Gunduz U (2015). Protein kinase D2 silencing reduced motility of doxorubicin-resistant MCF7 cells. Tumour Biol.

[88] Bernhart E, Damm S, Wintersperger A, Devaney T, Zimmer A, Raynham T (2013). Protein kinase D2 regulates migration and invasion of U87MG glioblastoma cells in vitro. Exp Cell Res.

[89] Hao Q, Wang L, Zhao ZJ, Tang H (2009). Identification of protein kinase D2 as a pivotal regulator of endothelial cell proliferation, migration, and angiogenesis. J Biol Chem.

[90] Tan M, Hao F, Xu X, Chisolm GM, Cui MZ (2009). Lysophosphatidylcholine activates a novel PKD2-mediated signaling pathway that controls monocyte migration. Arterioscler Thromb Vasc Biol.

[91] Döppler H, Bastea LI, Borges S, Spratley SJ, Pearce SE, Storz P (2014). Protein kinase D isoforms differentially modulate cofilin-driven directed cell migration. PLoS One.

[92] Christoforides C, Rainero E, Brown KK, Norman JC, Toker A (2012). PKD controls alphavbeta3 integrin recycling and tumor cell invasive migration through its substrate rabaptin-5. Dev Cell.

[93] Eiseler T, Wille C, Koehler C, Illing A, Seufferlein T (2016). Protein kinase D2 assembles a multiprotein complex at the trans-golgi network to regulate matrix metalloproteinase secretion. J Biol Chem.

[94] Lee S, Chung CY (2009). Role of VASP phosphorylation for the regulation of microglia chemotaxis via the regulation of focal adhesion formation/maturation. Mol Cell Neurosci.

[95] Eiseler T, Hausser A, De Kimpe L, Van Lint J, Pfizenmaier K (2010). Protein kinase D controls actin polymerization and cell motility through phosphorylation of cortactin. J Biol Chem.

[96] Azoitei N, Pusapati GV, Kleger A, Moller P, Kufer R, Genze F (2010). Protein kinase D2 is a crucial regulator of tumour cell-endothelial cell communication in gastrointestinal tumours. Gut.

[97] Matthews SA, Navarro MN, Sinclair LV, Emslie E, Feijoo-Carnero C, Cantrell DA (2010). Unique functions for protein kinase D1 and protein kinase D2 in mammalian cells. Biochem J.

[98] Kim YI, Park JE, Brand DD, Fitzpatrick EA, Yi AK (2010). Protein kinase D1 is essential for the proinflammatory response induced by hypersensitivity pneumonitis-causing thermophilic actinomycetes saccharopolyspora rectivirgula. J Immunol.

[99] Mazzeo C, Calvo V, Alonso R, Merida I, Izquierdo M (2016). Protein kinase D1/2 is involved in the maturation of multivesicular bodies and secretion of exosomes in T and B lymphocytes. Cell Death Differ.

[100] Navarro MN, Goebel J, Hukelmann JL, Cantrell DA. Quantitative phosphoproteomics of cytotoxic T cells to reveal protein kinase D 2 regulated networks. Mol Cell Proteomics. 2014; doi: 10.1074/mcp.M113.037242. 10.1074/mcp.M113.037242 PMC4256504 25266776

[101] Chung SM, Bae ON, Lim KM, Noh JY, Lee MY, Jung YS (2007). Lysophosphatidic acid induces thrombogenic activity through phosphatidylserine exposure and procoagulant microvesicle generation in human erythrocytes. Arterioscler Thromb Vasc Biol.

[102] Prada I, Furlan R, Matteoli M, Verderio C (2013). Classical and unconventional pathways of vesicular release in microglia. Glia.

[103] Biber K, Owens T, Boddeke E (2014). What is microglia neurotoxicity (not)?. Glia.

[104] Liu B, Gao HM, Wang JY, Jeohn GH, Cooper CL, Hong JS (2002). Role of nitric oxide in inflammation-mediated neurodegeneration. Ann N Y Acad Sci.

[105] Hsieh HL, Yang CM (2013). Role of redox signaling in neuroinflammation and neurodegenerative diseases. Biomed Res Int.

[106] Saha RN, Pahan K (2006). Regulation of inducible nitric oxide synthase gene in glial cells. Antioxid Redox Signal.

[107] Kumar A, Chen SH, Kadiiska MB, Hong JS, Zielonka J, Kalyanaraman B (2014). Inducible nitric oxide synthase is key to peroxynitrite-mediated, LPS-induced protein radical formation in murine microglial BV2 cells. Free Radic Biol Med.

[108] Valko M, Leibfritz D, Moncol J, Cronin MT, Mazur M, Telser J (2007). Free radicals and antioxidants in normal physiological functions and human disease. Int J Biochem Cell Biol.

[109] Hambardzumyan D, Bergers G (2015). Glioblastoma: defining tumor niches. Trends Cancer.

[110] Hambardzumyan D, Gutmann DH, Kettenmann H (2016). The role of microglia and macrophages in glioma maintenance and progression. Nat Neurosci.

[111] Poon CC, Sarkar S, Yong VW, Kelly JJ. Glioblastoma-associated microglia and macrophages: targets for therapies to improve prognosis. Brain. 2017; doi: 10.1093/brain/aww355. 10.1093/brain/aww355 28334886

[112] Brennan CW, Verhaak RG, McKenna A, Campos B, Noushmehr H, Salama SR (2013). The somatic genomic landscape of glioblastoma. Cell.

[113] Hoelzinger DB, Mariani L, Weis J, Woyke T, Berens TJ, McDonough WS (2005). Gene expression profile of glioblastoma multiforme invasive phenotype points to new therapeutic targets. Neoplasia.

[114] Szulzewsky F, Pelz A, Feng X, Synowitz M, Markovic D, Langmann T (2015). Glioma-associated microglia/macrophages display an expression profile different from M1 and M2 polarization and highly express Gpnmb and Spp1. PLoS One.

[115] Zhang J, Sarkar S, Cua R, Zhou Y, Hader W, Yong VW (2012). A dialog between glioma and microglia that promotes tumor invasiveness through the CCL2/CCR2/interleukin-6 axis. Carcinogenesis.

