# Supplementary figures and images for: Lysophosphatidic acid via LPA-receptor 5/protein kinase D-dependent pathways induces a motile and pro-inflammatory microglial phenotype
Source: J Neuroinflammation. 2017 Dec 19;14:253. doi: 10.1186/s12974-017-1024-1 (PMC5735906; doi:10.1186/s12974-017-1024-1)

## Slide 1
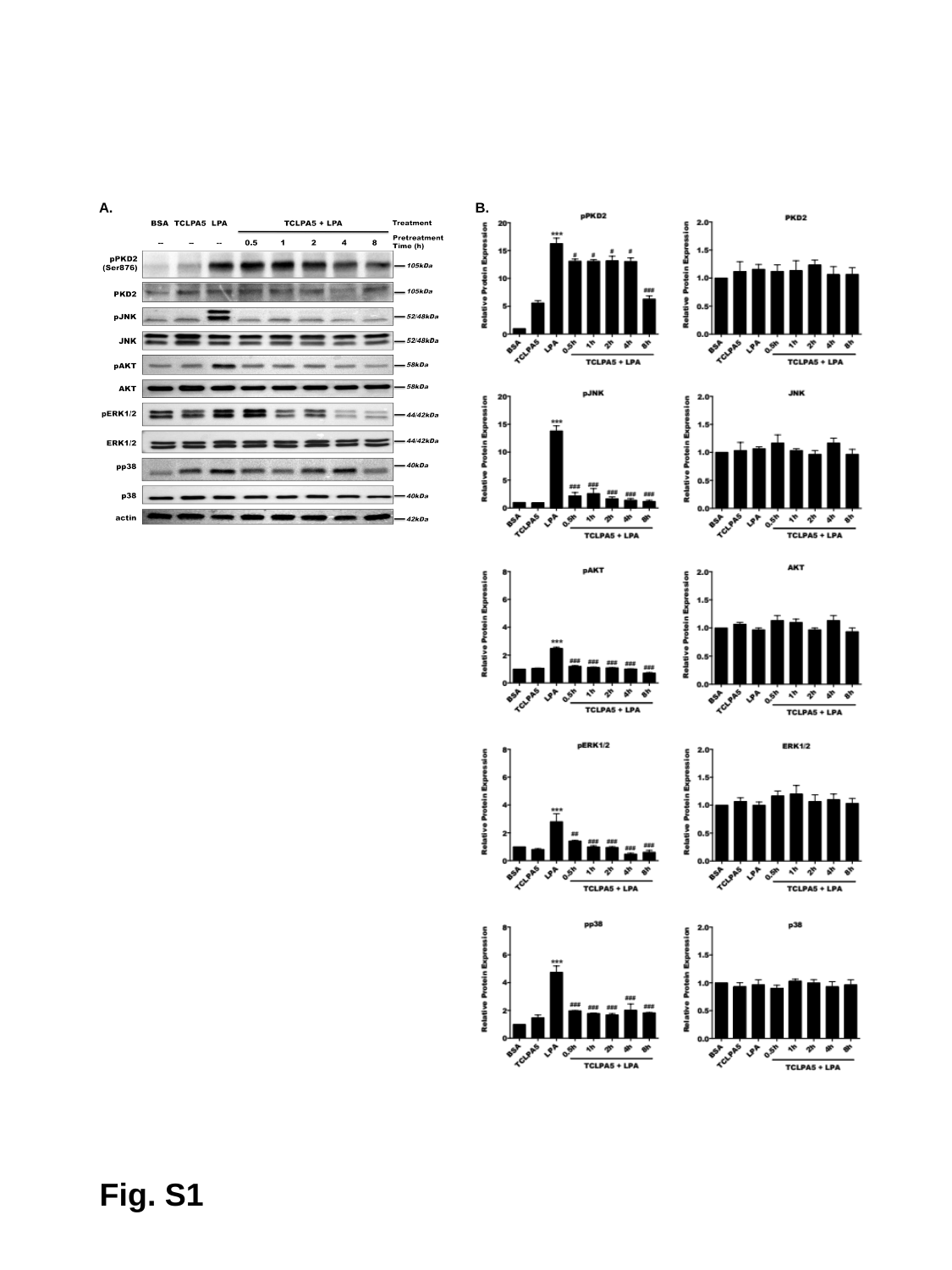

B.
A.
Fig. S1

Supplement: Supplementary file 1 — LPAR5 controls LPA-mediated downstream signaling. (A) BV-2 microglia cells were cultured in 6-well plates and serum-starved overnight. The cells were preincubated with TCLPA5 (5 μM) for the indicated times and then incubated with LPA (1 μM) or LPA (1 μM) plus TCLPA5. Cells incubated only with 0.1% BSA or TCLPA5 (5 μM) were used as negative control. The phosphorylation states of PKDs, JNK, AKT, ERK1/2, and p38 were detected using western blotting. One representative blot is shown. (B) Densitometric analysis of western blots (N = 3). Results represent mean values + SEM (***p < 0.001 compared to control; ## p < 0.01, ### p < 0.001 LPA plus TCLPA5 versus LPA; one-way ANOVA with Bonferroni correction). (PPT 846 kb) [file 12974_2017_1024_MOESM1_ESM.ppt]

## Slide 1
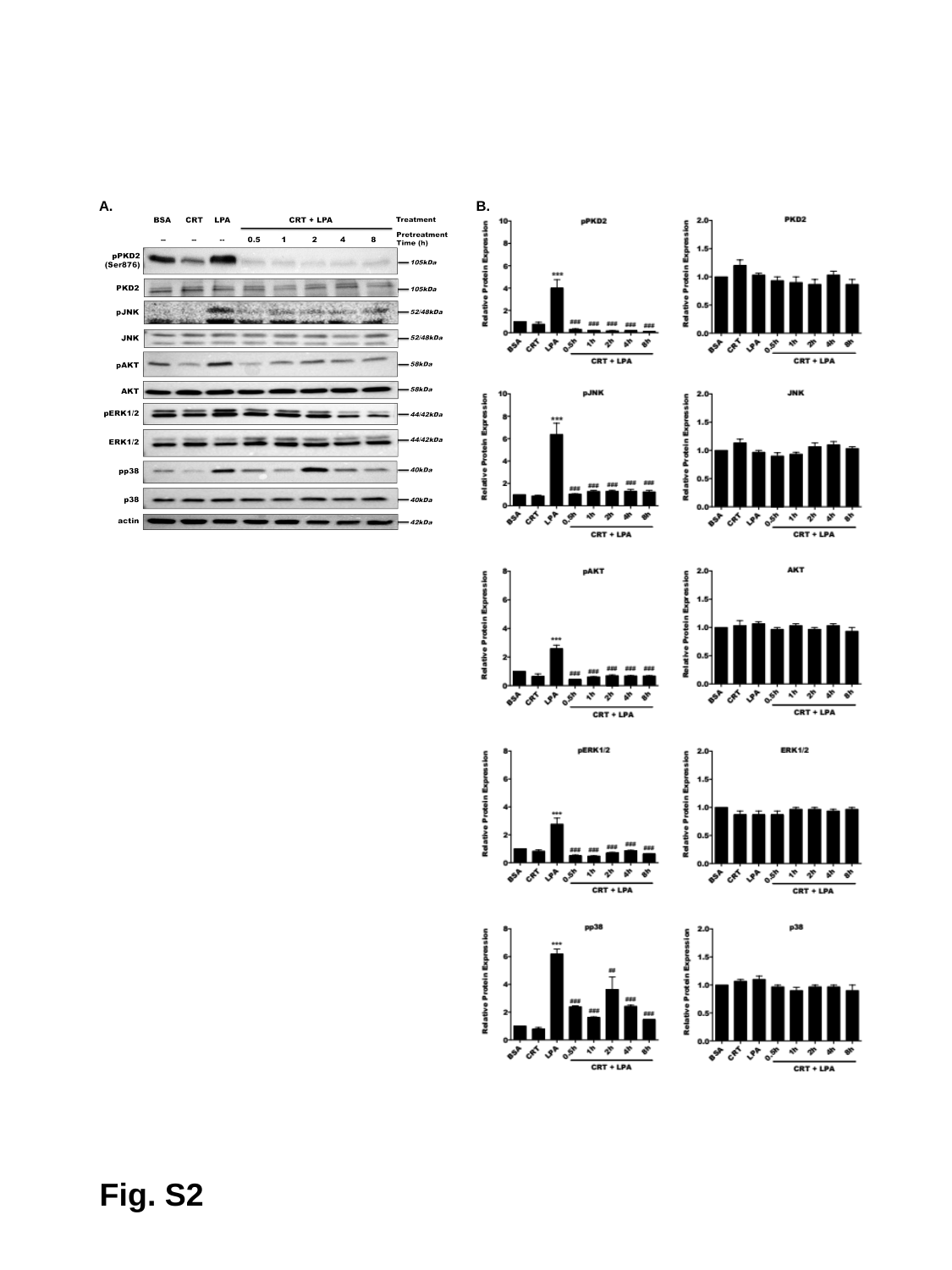

B.
A.
Fig. S2

Supplement: Supplementary file 2 — PKD family inhibition abrogates LPA-mediated downstream signaling. (A) BV-2 microglia cells were cultured in 6-well plates, serum-starved overnight and preincubated with CRT0066101 (‘CRT’, 1 μM) for the indicated time periods before incubation with LPA (1 μM) or LPA (1 μM) plus CRT. Cells incubated only with 0.1% BSA or CRT (1 μM) were used as negative control. The phosphorylation states of PKDs, JNK, AKT, ERK1/2, and p38 were detected by immunoblotting and one representative blot for each protein is shown. (B) Densitometric analysis of western blots (N = 3). Results are presented as mean values + SEM (***p < 0.001; compared to control; ## p < 0.01, ### p < 0.001 LPA plus CRT versus LPA; one-way ANOVA with Bonferroni correction). (PPT 777 kb) [file 12974_2017_1024_MOESM2_ESM.ppt]

## Slide 1
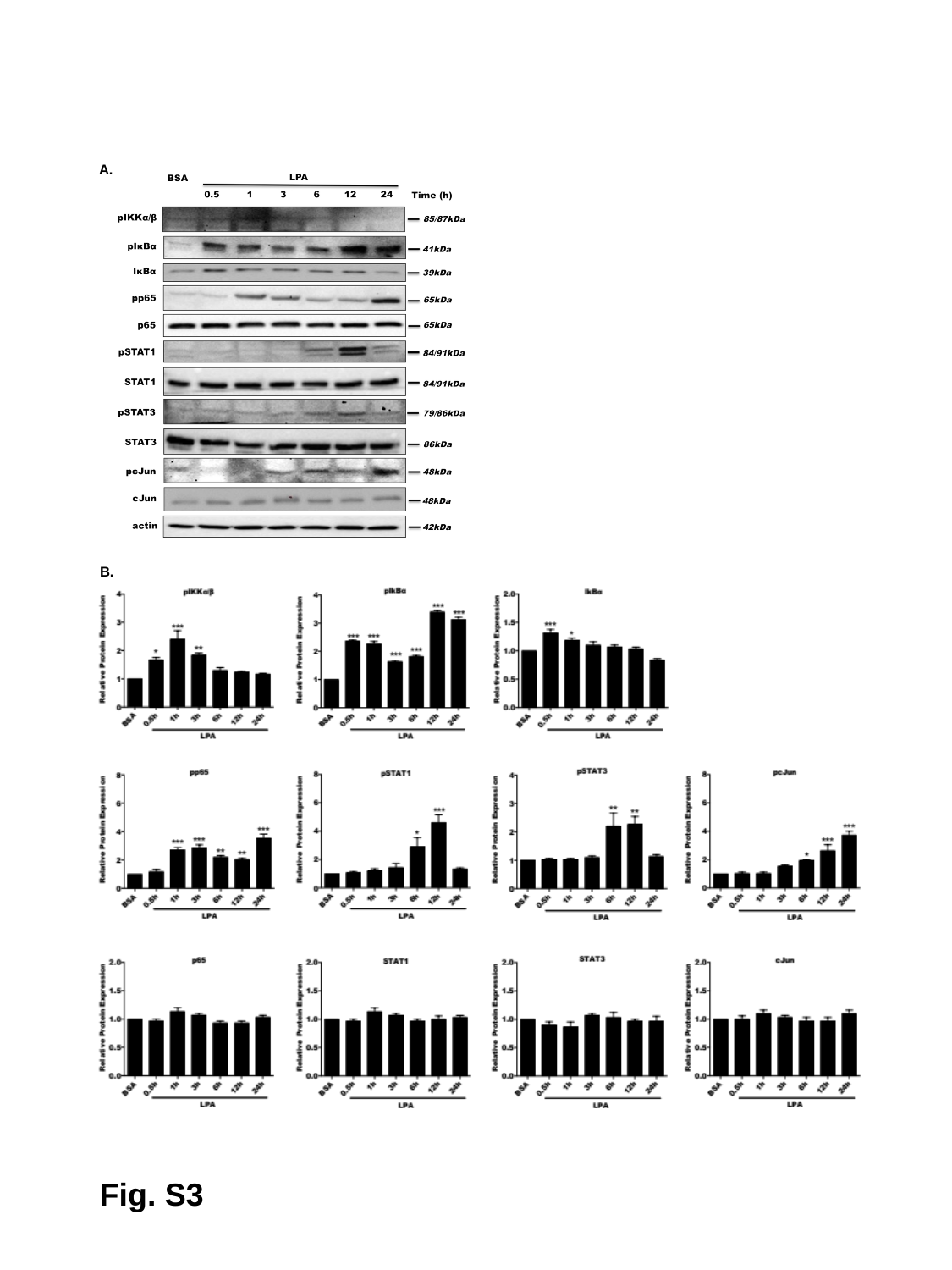

A.
B.
Fig. S3

Supplement: Supplementary file 3 — LPA promotes activation of pro-inflammatory transcription factors in BV-2 cells. Serum-starved (A) BV-2 cells were treated with 0.1% BSA (control) or LPA (1 μM) for the indicated time periods, the cellular protein lysates were collected and phosphorylation state of IKKα/β, IκBα, p65-NF-κB, STAT1, STAT3, and c-Jun was detected using immunoblotting. One representative blot out of N = 3 experiments is shown. Actin was used as loading control. (B) Densitometric analysis of western blots show the significance of changes in the protein expression and represent mean values + SEM (*p < 0.05, **p < 0.01, ***p < 0.001; one-way ANOVA with Bonferroni correction). (PPT 582 kb) [file 12974_2017_1024_MOESM3_ESM.ppt]

## Slide 1
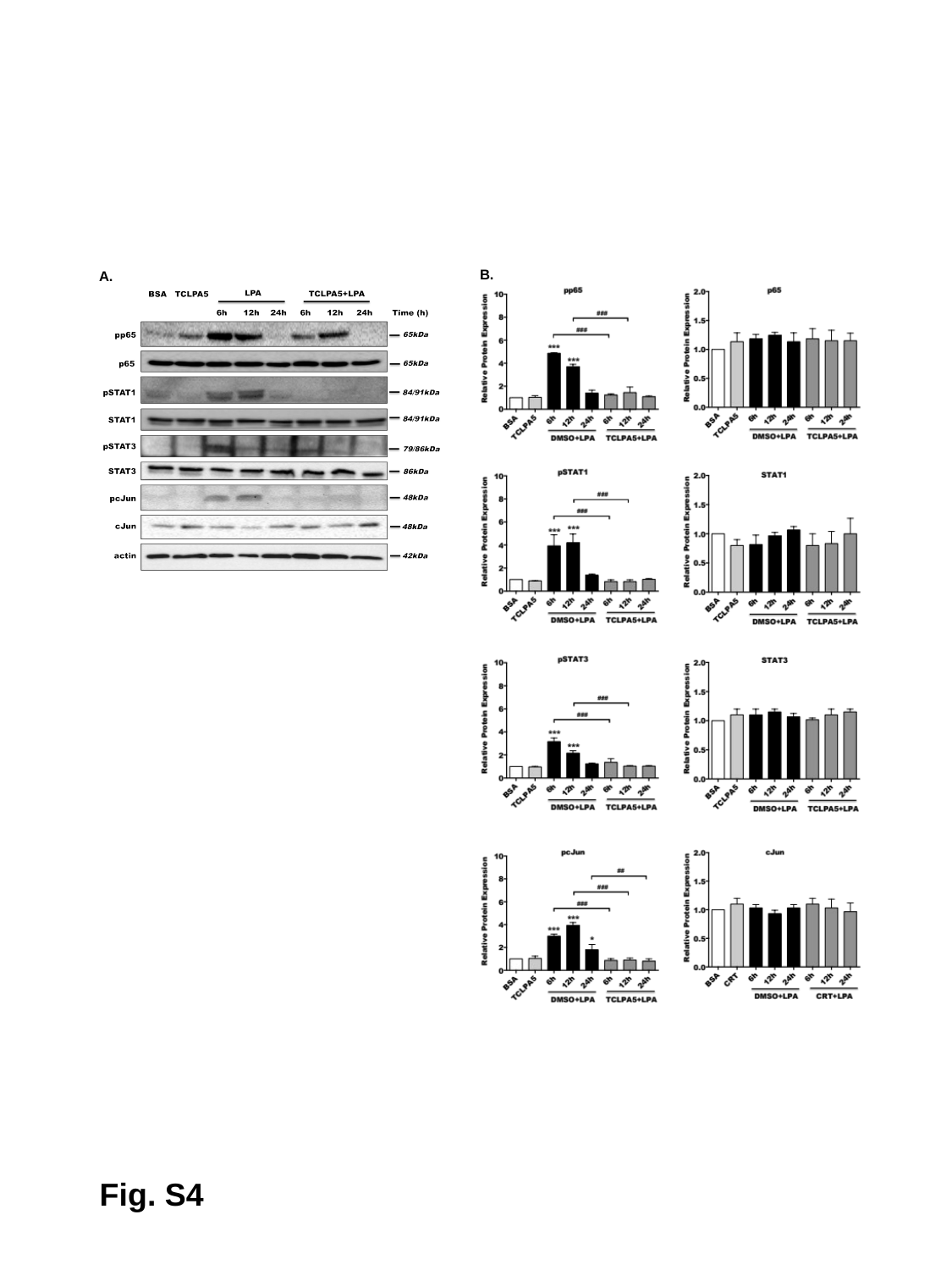

B.
A.
Fig. S4

Supplement: Supplementary file 4 — TCLPA5 inhibits the phosphorylation of pro-inflammatory transcription factors. BV-2 microglia cells were cultured in 6-well plates and serum-starved overnight. Cells were treated with LPA (1 μM) or LPA (1 μM) in the presence of (A) TCLPA5 (5 μM) for the indicated time periods. Cells incubated only with 0.1% BSA or TCLPA5 (5 μM) were used as negative control. The phosphorylation of p65-NF-κB, STAT1, STAT3, and c-Jun was detected using western blotting and one representative blot is shown. (B) Densitometric analysis of western blots (N = 3). Results represent mean values + SEM (*p < 0.05, ***p < 0.001 compared to control; ## p < 0.01, ### p < 0.001 LPA plus TCLPA5 versus LPA; one-way ANOVA with Bonferroni correction). (PPT 511 kb) [file 12974_2017_1024_MOESM4_ESM.ppt]

## Slide 1
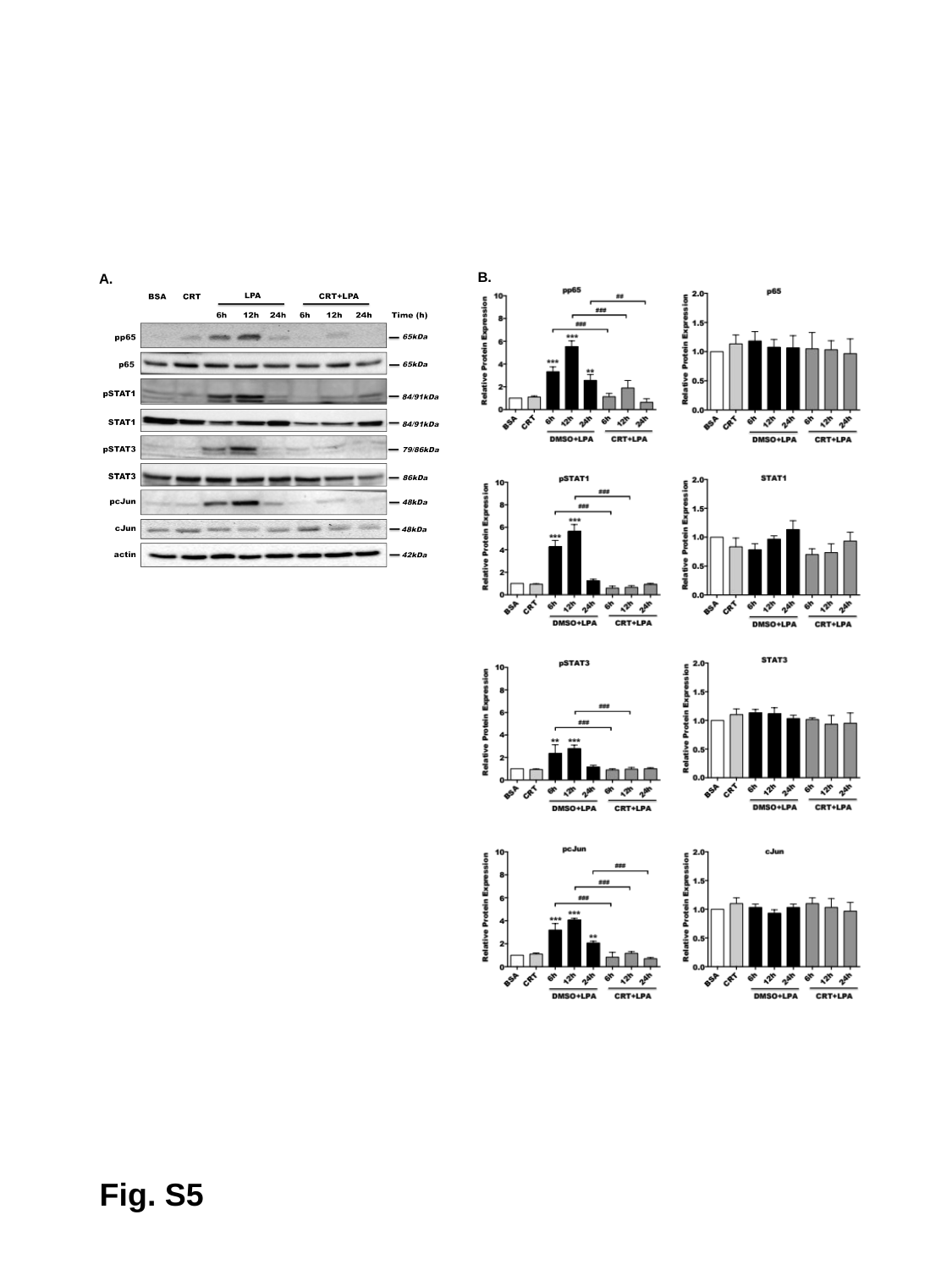

B.
A.
Fig. S5

Supplement: Supplementary file 5 — The phosphorylation of pro-inflammatory transcription factors is under PKD family control. BV-2 cells, serum-starved overnight and treated with LPA (1 μM) or LPA (1 μM) in the presence of (A) CRT0066101 (1 μM) for the indicated time periods. Cells incubated only with 0.1% BSA or CRT (1 μM) were used as negative control. The phosphorylation state of p65-NF-κB, STAT1, STAT3, and c-Jun was detected by western blotting. One representative blot is shown. (B) Densitometric analysis of western blots (N = 3). Results are presented as mean values + SEM (**p < 0.01, ***p < 0.001 compared to control; ## p < 0.01, ### p < 0.001 LPA plus CRT versus LPA; one-way ANOVA with Bonferroni correction). (PPT 500 kb) [file 12974_2017_1024_MOESM5_ESM.ppt]

## Slide 1
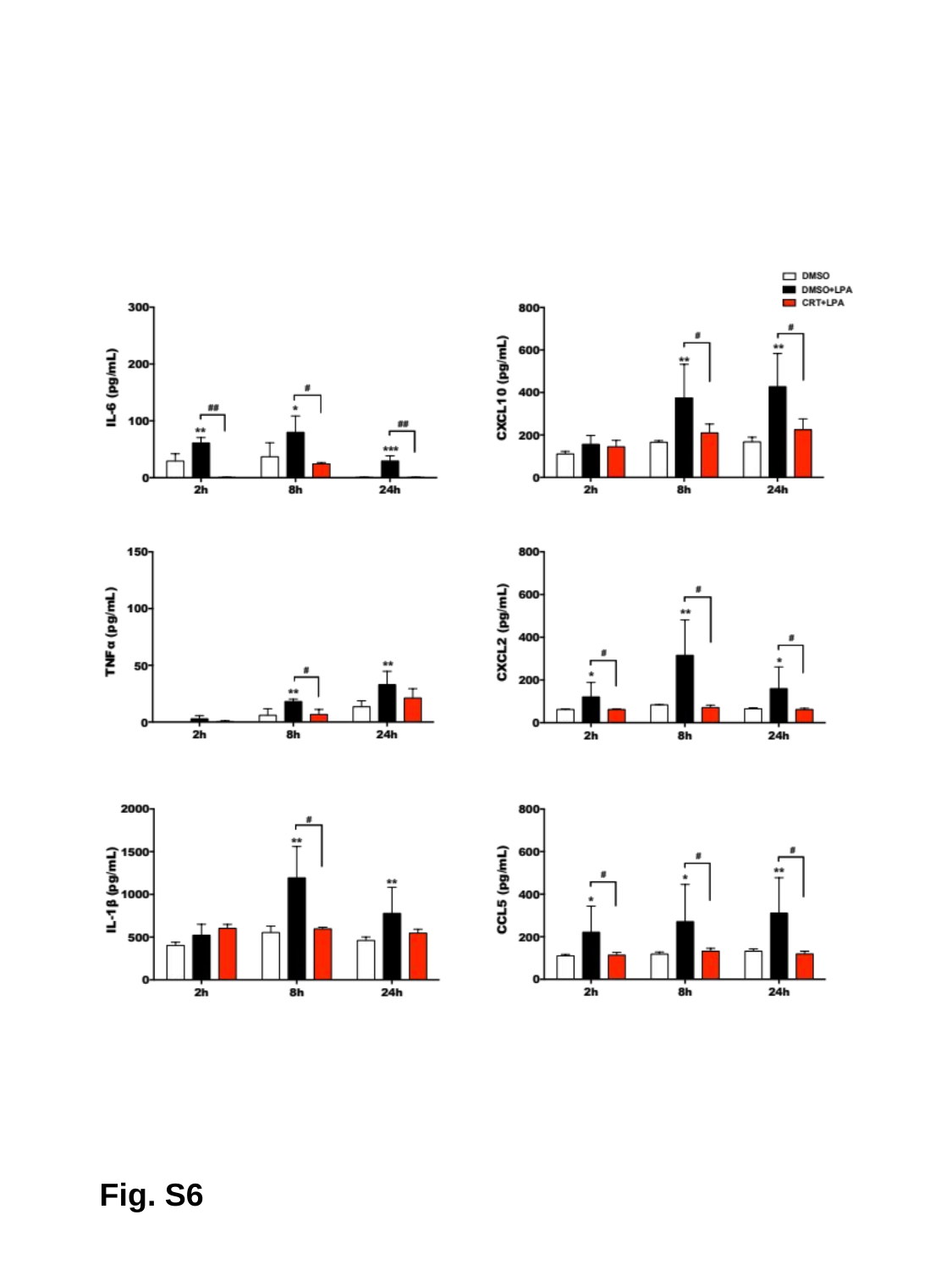

Fig. S6

Supplement: Supplementary file 6 — PKD family members control the secretion of pro-inflammatory cytokines and chemokines. BV-2 cells were cultured on 12-well plates; serum-starved o/n and the supernatants were collected after incubation with DMSO, DMSO plus LPA (1 μM) or LPA (1 μM) plus CRT (1 μM). ELISAs were used to quantitate IL-6, IL-1β, CXCL10 (IP-10), TNF-α, CXCL2 (MIP-2), and CCL5 (RANTES) concentrations. Results shown represent mean + SD from three independent experiments performed in triplicate (*p < 0.05; **p < 0.01 compared to vehicle control; # p < 0.05, ## p < 0.01; CRT + LPA compared to LPA treated cells; one-way ANOVA with Bonferroni correction). (PPT 174 kb) [file 12974_2017_1024_MOESM6_ESM.ppt]
